# Loss of Carbamoyl Phosphate Synthetase 1 Potentiates Hepatocellular Carcinoma Metastasis by Reducing Aspartate Level

**DOI:** 10.1002/advs.202402703

**Published:** 2024-10-10

**Authors:** Siyuan Chen, Qin Tang, Manqiu Hu, Sijie Song, Xiaohong Wu, You Zhou, Zihan Yang, Siqi Liao, Li Zhou, Qingliang Wang, Hongtao Liu, Mengsu Yang, Zhe‐Sheng Chen, Wei Zhao, Song He, Zhihang Zhou

**Affiliations:** ^1^ Department of Gastroenterology The Second Affiliated Hospital of Chongqing Medical University Chongqing 400010 China; ^2^ Department of Biomedical Sciences and Tung Biomedical Sciences Center City University of Hong Kong 83 Tat Chee Avenue Kowloon Hong Kong SAR 999077 P. R. China; ^3^ Department of Pathology The Second Affiliated Hospital of Chongqing Medical University Chongqing 400010 China; ^4^ Department of Pharmaceutical Sciences Institute for Biotechnology College of Pharmacy and Health Sciences St. John's University Queens NY 11439 USA; ^5^ School of Clinical Medicine The First Affiliated Hospital Chengdu Medical College Sichuan 610500 P. R. China; ^6^ Department of Clinical Biochemistry School of Laboratory Medicine Chengdu Medical College Sichuan 610500 P. R. China

**Keywords:** aspartate, CPS1, m6A, metastasis, PC‐PLC

## Abstract

Hepatocellular carcinoma (HCC) is one of the most lethal cancers worldwide. Numerous studies have shown that metabolic reprogramming is crucial for the development of HCC. Carbamoyl phosphate synthase 1 (CPS1), a rate‐limiting enzyme in urea cycle, is an abundant protein in normal hepatocytes, however, lacking systemic research in HCC. It is found that CPS1 is low‐expressed in HCC tissues and circulating tumor cells, negatively correlated with HCC stage and prognosis. Further study reveals that CPS1 is a double‐edged sword. On the one hand, it inhibits the activity of phosphatidylcholine‐specific phospholipase C to block the biosynthesis of diacylglycerol (DAG), leading to the downregulation of the DAG/protein kinase C pathway to inhibit invasion and metastasis of cancer cells. On the other hand, CPS1 promotes cell proliferation by increasing intracellular S‐adenosylmethionin to enhance the m6A modification of solute carrier family 1 member 3 mRNA, a key transporter for aspartate intake. Finally, CPS1 overexpressing adeno‐associated virus can dampen HCC progression. Collectively, this results uncovered that CPS1 is a switch between HCC proliferation and metastasis by increasing intracellular aspartate level.

## Graphical abstract

This study reveals that CPS1 expression is reduced in HCC and has a dual regulatory effect on HCC. Loss of CPS1 inhibits cell proliferation, but facilitates the metastasis of HCC. CPS1 upregulates SLC1A3 by m6A modification to enhance Asp import thus fueling cell proliferation and meanwhile inhibiting the PC‐PLC/DAG/PKC axis.

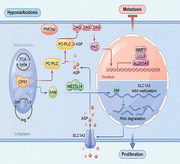

## 1 Introduction

Primary liver cancer is the fourth leading cause of cancer‐related death worldwide, among which hepatocellular carcinoma (HCC) is the main type.^[^ ^1^, ^2^, ^3^ ^]^ Given that there are many limitations both in surgery and targeted drugs in the treatment of HCC, it is important to explore the underlying mechanisms of HCC progression and identify molecular targets for early diagnosis or treatment. The occurrence and progression of cancer are accompanied by complex metabolic reprogramming to ensure sufficient nutrient and energy supply.^[^ ^4^, ^5^, ^6^ ^]^ Numerous studies have shown that metabolic reprogramming is a hallmark of cancers. Among them, glucose metabolism,^[^ ^7^, ^8^ ^]^ lipid metabolism,^[^ ^9^ ^]^ and amino acid metabolism^[^ ^10^ ^]^ are the three main fields, which can transform to each other through the tricarboxylic acid cycle (TCA cycle), forming a large and complicated regulatory system.

Liver is the main organ for biological detoxification, in which toxic ammonia is transformed into urea through urea cycle. Carbamoyl phosphate synthase 1 (CPS1) is the rate limiting enzyme in the first step of the urea cycle, and is the most abundant mitochondrial matrix protein in normal liver cells.^[^ ^11^, ^12^ ^]^ The urea cycle produces arginine (Arg), citrulline (Cit), ornithine (Orn), and consumes aspartate (Asp), which can be used in metabolic pathways such as the TCA cycle and nucleotide synthesis.^[^ ^13^ ^]^ In the past years, a small number of studies found that CPS1 is involved in the formation and development of tumors. In non‐small cell lung cancer, CPS1 promotes cell proliferation and inhibits apoptosis by increasing pyrimidine synthesis.^[^ ^14^ ^]^ CPS1 expression level is increased in intrahepatic cholangiocarcinoma,^[^ ^15^ ^]^ colorectal cancer,^[^ ^16^, ^17^ ^]^ glioblastoma^[^ ^18^ ^]^ and bladder cancer,^[^ ^19^ ^]^ which promote cell proliferation, and is negatively associated with the prognosis of patients. Wheeler et al. found that the expression of CPS1 was reduced in HCC due to methylation on the promoter.^[^ ^20^ ^]^ Chen et al. also found this phenomenon in more than 10 000 liver tissues of HCC patients, and further demonstrated that lack of CPS1 could promote stemness of HCC cells by affecting fatty acid metabolism.^[^ ^12^ ^]^ Besides, Xue et al. revealed that CPS1 deficiency could accelerate the development of HCC and induce radiation resistance in HCC both in vitro and in vivo.^[^ ^21^ ^]^ However, the exact role and mechanism of CPS1 in HCC metabolic reprogramming is not clear and merits further study.

It is noteworthy that, Asp is used as a drug to treat hepatic encephalopathy in clinics by enhancing the urea cycle. However, the function of Asp in tumors are controversial. On the one hand, some studies declared Asp promoted cell proliferation,^[^ ^22^, ^23^, ^24^ ^]^ and Asp enhanced tumor cell metastasis in melanoma by disrupting tumor‐associated metabolism of nucleotide synthesis and glycosylation.^[^ ^25^, ^26^ ^]^ On the other hand, Ochocki et al. proved that overexpression of solute carrier family 1 member 3 (SLC1A3, a key Asp transporter) or increase Asp inhibited proliferation of clear cell renal cell carcinoma.^[^ ^27^ ^]^ However, the regulation of Asp on liver cancer metastasis is unclear.

Our study showed that CPS1 was downregulated in HCC tissues. Negative expression of CPS1 in HCC was correlated with advanced tumor stage and poor patient prognosis. Similarly, CPS1 expression was low or negative in circulating tumor cells (CTCs) of HCC patients and negatively associated with EpCAM expression. We further revealed the double‐faced function of CPS1 in HCC by regulating intracellular Asp level. On the one hand, CPS1 increased the intracellular Asp by enhancing the m6A modification of SLC1A3, to fuel HCC cell proliferation. On the other hand, the CPS1‐induced upregulation of Asp could prevent invasion and metastasis of HCC cells by inhibiting the activity of phosphatidylcholine specific phospholipase C (PC‐PLC) to block the biosynthesis of diacylglycerol (DAG), leading to the downregulation of DAG‐PKC (protein kinase C) axis, which have been shown to promote invasion and metastasis of various cancer cells. Herein, we demonstrate that CPS1 acts as a switch between HCC proliferation and metastasis by increasing intracellular Asp content, reflecting the dynamic needs for amino acids during cancer progression.

## 2 Results

### 2.1 Negative CPS1 Expression was Associated with Advanced HCC Stage

**Figure 1:**
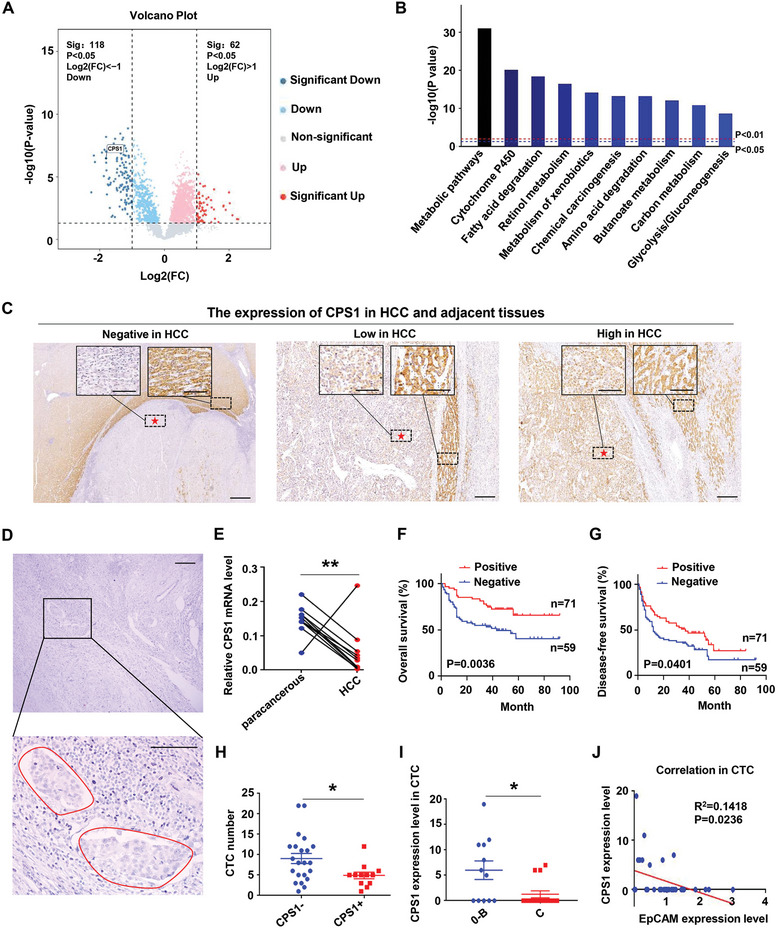
Reduced expression of CPS1 in HCC. A) Proteomics detection of differentially expressed proteins between HCC and adjacent tissues, *n* = 8. B) Proteomics KEGG analysis of changes in cellular pathways. C) immunohistochemical (IHC) detection of CPS1 expression in HCC and adjacent tissues, *n* = 152. The red five‐pointed star indicates tumor area. D) The expression of CPS1 in cancer thrombi. E) qPCR detection of mRNA levels of CPS1 in HCC and adjacent tissues, *n* = 10 in each group. F, G) Overall survival analysis and disease‐free survival analysis of patients with negative and positive expression of CPS1 expression. H) Number of circulating tumor cells (CTC) in patients with negative and positive CPS1 expression. CPS1^−^: *n* = 22, CPS1^+^: *n* = 12. I) The expression level of CPS1 in CTC of HCC patients with different Barcelona (BCLC) stages. BCLC 0‐II: *n* = 12, BCLC III: *n* = 15. J) Correlation analysis between CPS1 expression and EpCAM expression. Scale bar: 50 × magnification, 400 µm, 200 × magnification, 100 µm. Values represent the means±SDs. *: *p* < 0.05, **: *p* < 0.01.

To explore the protein changes during the development of liver cancer, we collected 8 cases of HCC tissues and corresponding adjacent tissues for proteomic analysis. The results showed that, compared with adjacent tissues, the protein level of CPS1 in HCC tissues decreased dramatically (**Figure** **1**A). KEGG analysis displayed changes in metabolic pathways were most pronounced (Figure 1B). In addition, CPS1 has three different expression patterns in liver cancer (negative expression, low expression, and high expression, Figure 1C,D). Utilizing 10 HCC tissues and adjacent tissues, we found that the mRNA expression of CPS1 was also reduced in HCC (Figure 1E). Through clinical pathological analysis, we revealed that negative CPS1 expression was significantly correlated with tumor size, macrovascular invasion, Bacelona Clinic Liver Cancer (BCLC) stage and poor differentiation (**Table** **1**). Survival analysis showed that the positive expression of CPS1 was correlated with longer overall and disease‐free survival time (Figure 1F,G). Because CPS1 expression is negatively associated with vascular invasion, we collected CTCs from 34 HCC patients, and found that the patients with negative CPS1 expression had more CTCs (Figure 1H). Besides, CPS1 protein intensity was significantly lower in CTC of BCLC‐C patients than that in patients with phase 0‐B (Figure 1I). The expression of CPS1 was also negatively associated with the expression of epithelial marker EpCAM in CTCs (Figure 1J). These results indicated that reduced expression of CPS1 in HCC could promote deterioration.

**Table 1:** The correlation between CPS1 expression level with clinicopathological characteristics in HCC patients.

Clinicopathologic features	CPS1 expression		*p* value
	Negative (n = 85) No.patient [%]	Positive (n = 67) No.patient [%]	
**Gender**			0.389
Male	73 (85.9)	54 (80.6)	
Female	12 (14.1)	13 (19.4)	
**Age**			0.745
≤52	47 (55.3)	35 (52.2)	
>52	38 (44.7)	32 (47.8)	
**Number**			0.195
1	73 (85.9)	62 (92.5)	
2	9 (10.6)	2 (3)	
3	3 (3.5)	3 (4.5)	
**Size (cm)**			0.002
≤3	26 (30.6)	38 (56.7)	
>3	59 (69.4)	29 (43.3)	
**Macrovascular invasion**			0.036
No	68 (80.0)	62 (92.5)	
Yes	17 (20.0)	5 (7.5)	
**Distant metastasis**			0.348
No	77 (90.6)	64 (95.5)	
Yes	8 (9.4)	3 (4.5)	
**Child‐Pugh stage**			0.404
1	78 (91.8)	59 (88.1)	
2	6 (7.1)	8 (11.9)	
3	1 (1.2)	0 (0.0)	
**BCLC stage**			0.024
0/A/B	66 (77.6)	61 (91.0)	
C	19 (22.4)	6 (9.0)	
**Differentiation**			0.006
Poor	15 (17.6)	7 (10.6)	
Medium	63 (74.1)	41 (62.1)	
Well	7 (8.2)	18 (27.3)	
**Microinvasion**			0.631
No	82 (96.5)	66 (98.5)	
Yes	3 (3.5)	1 (1.5)	
**Ki67 level**			0.137
Low	55 (64.7)	35 (52.2)	
High	30 (35.3)	32 (47.8)	
**PS score**			0.334
0	68 (80.0)	49 (73.1)	
1	15 (17.6)	12 (17.9)	
2	1 (1.2)	6 (9.0)	
3	1 (1.2)	0 (0.0)	

The statistical method adopts the Two tailed test.

### 2.2 Loss of CPS1 Promoted HCC Metastasis

CPS1 expression was detected in various human HCC cell lines (Huh7, PLC/PRF/5 (PLC), SMMC‐7721 (7721), HepG2, SK‐Hep1 (SK)) and a normal liver cell line LO2. The result of real‐time quantitative PCR (qPCR) showed that, compared with LO2 cells, CPS1 mRNA was expressed at the highest level in PLC cells. HepG2 and SK cells had the lowest CPS1 mRNA level (Figure S1A, Supporting Information). Immunoblotting and immunofluorescence exhibited the CPS1 protein expression was consistent with that of qPCR (Figure S1B,C, Supporting Information). CPS1 was knocked down with lentivirus infection in PLC and Huh7 cells. After screening, the interference sequences 641 and 642 were used for subsequent experiments to prepare stable CPS1 knockdown cell lines (PLC shCPS1‐1/2 and Huh7 shCPS1‐1/2). Adenovirus containing intact CPS1 coding sequence (CDS) infected SK cells were used to prepare CPS1 overexpression cell lines (SK CPS1‐OE). The corresponding control cells were used as negative controls (NC). The efficiency of virus infection was verified by qPCR and western blotting (Figure S1D–F, Supporting Information).

**Figure 2:**
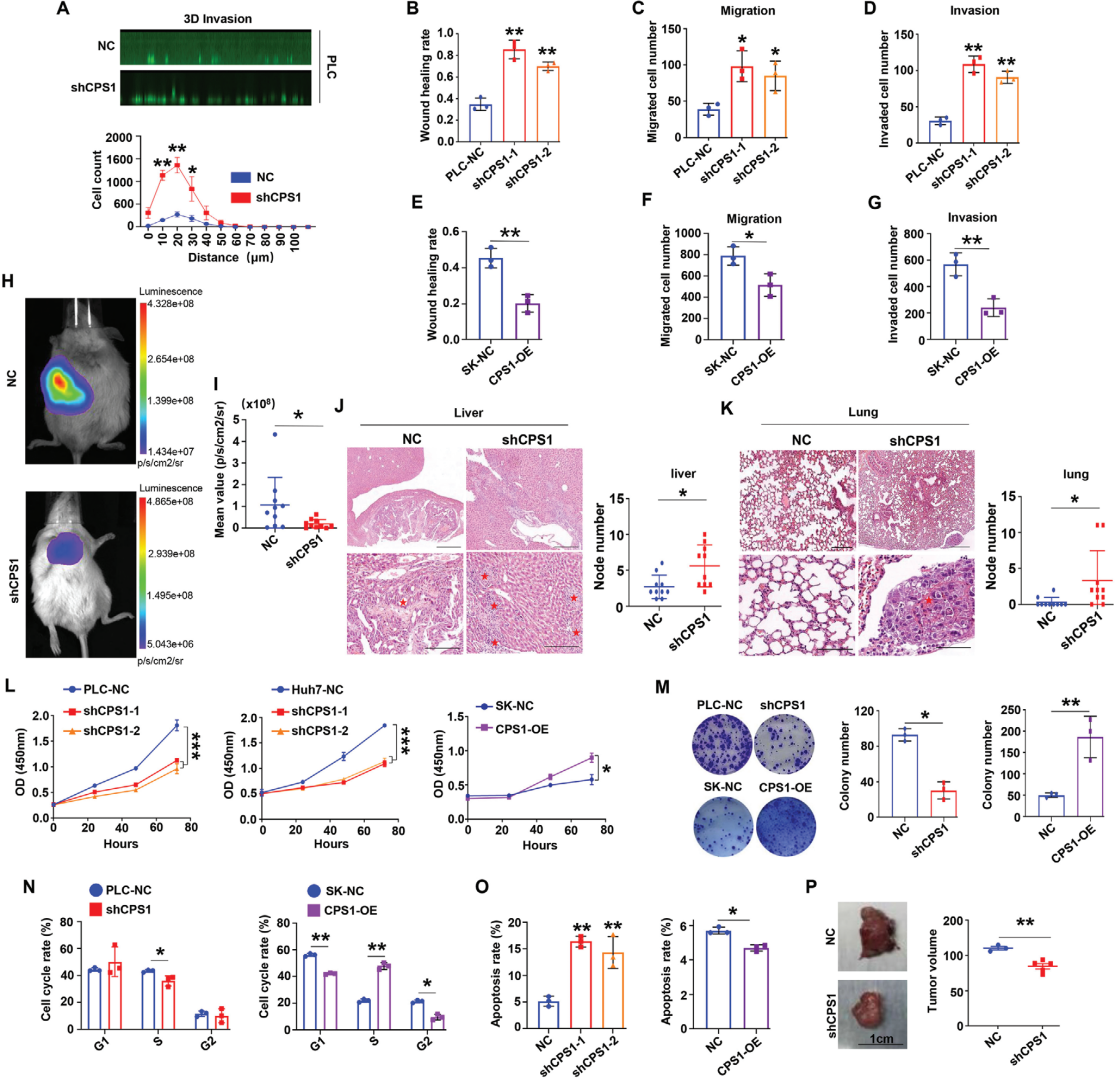
Knocking down the expression of CPS1 promotes HCC progression. A) 3D migration experiments on cell invasion in PLC‐NC and PLC‐shCPS1 cells. B–G) Statistical results of cell migration and invasion detected by wound healing and transwell experiment, respectively. H) In vivo fluorescence imaging shows the tumorigenesis status of the PLC‐NC group and PLC‐shCPS1 group in an in situ tumor model, *n* = 10. I) Statistics of the number in situ tumor cells. J, K) Hematoxylin‐eosin staining (HE) staining detection of liver and lung metastases in PLC‐NC and PLC‐shCPS1 groups, *n* = 10. The red five‐pointed star indicates tumor area. L) CCK8 assays showed cell proliferation in PLC‐NC/shCPS1, Huh7‐NC/shCPS1 and SK‐NC/CPS1‐OE cells. M) Colony formation experiment shows the effect of CPS1 on the tumorigenic ability of HCC cells. The left panel shows the colony image, and the right panel shows the summary data of colony number. N) Flow cytometry detection of CPS1 regulation on cell cycle. O) Cell apoptosis analysis by flow cytometry in shCPS1, CPS1‐OE cells and control cells. P) In vivo tumor formation in PLC‐NC and PLC‐shCPS1 groups demonstrated by orthotopic tumor transplantation in nude mice, *n* = 3–4. Scale bar: 50 × magnification, 400 µm, 200 × magnification, 100 µm. Values represent the means±SDs. *: *p* < 0.05, **: *p* < 0.01, ***: *p* < 0.001. The above in vitro experiments have all been biologically replicated (n = 3).

3D invasion experiment was used to detect the impact of CPS1 on cell invasion. Although cells were all running on the same track at the beginning (0 µm), the number of PLC‐shCPS1 cells was approximately three times that of PLC‐NC cells at a migration distance of 10–30 µm (**Figure** **2**A). The wound healing experiment and transwell assay proved that cell migration and invasion ability significantly increased after knocking‐down CPS1 expression (Figure 2B–D, Figure S2A,B,E–G, Supporting Information). Contrarily, the cell migration and invasion decreased after up‐regulated CPS1 expression (Figure 2E–G, Figure S2C,D,H–J, Supporting Information). HCC orthotopic transplantation tumor model revealed that knock‐down of CPS1 led to less number of tumor cells (Figure 2H–I). Besides, both intrahepatic nodules and pulmonary metastases nodules increased significantly in shCPS1 group (Figures 2J,K and S2K,L, Supporting Information). Liver weight ratio reduced (Figure S2L, Supporting Information), however, there was no significant change in weight between shCPS1 group and NC group (Figure S2M, Supporting Information). The above results indicated that CPS1 inhibits the invasion and metastasis of HCC cells.

### 2.3 CPS1 Enhanced the Proliferation of HCC Cells

The clinical characteristics of CPS1 and the analysis of mice in situ liver tumor model hinted CPS1 might have double‐faced role in HCC as knocking down CPS1 led to reduce tumor volume but increase the number of metastatic foci. CCK8 assay revealed that when CPS1 expression was decreased, the proliferation of PLC cells and Huh7 cells was significantly reduced, while overexpression of CPS1 facilitated the proliferation of SK cells (Figure 2L). The colony formation experiment showed colony number of shCPS1 group was about half that of the NC group, while the CPS1‐OE cell colonies were three times that of the NC group (Figure 2M). Moreover, after knocking down CPS1, the number of S‐phase cells decreased and apoptosis ratio increased, while overexpression of CPS1 increased the number of S‐phase cells and decreased apoptosis ratio (Figure 2N,O, Figure S3, Supporting Information). Subcutaneous transplantation of tumor cells revealed the proliferative effect of CPS1 in vivo (Figure 2P). The above results reflected that CPS1 promote proliferation of HCC cells.

### 2.4 CPS1 Up‐Regulated SLC1A3 to Facilitate Asp Intake in HCC Cells

**Figure 3:**
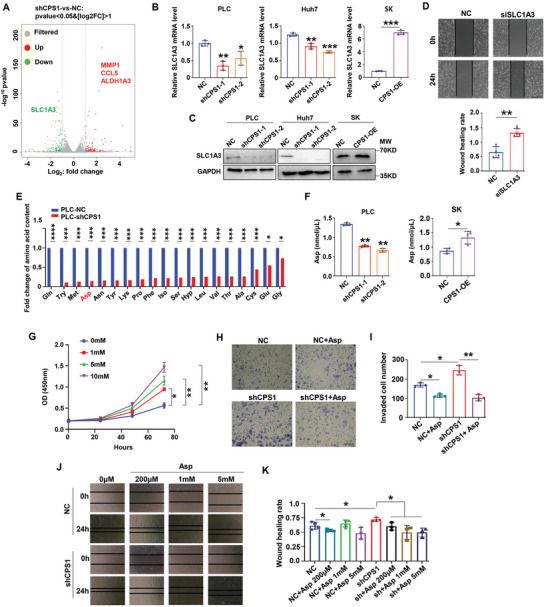
CPS1 increases the expression of SLC1A3 and regulates tumor cell proliferation, invasion, and metastasis through Asp. A) Transcriptome sequencing reveals genes affected by CPS1 (n = 3). B, C) qPCR and WB detection of the regulatory effect of CPS1 on SLC1A3 expression. D) Effect of SLC1A3 on HCC cell migration detected by wound healing experiment. The upper panel displays the wound healing images, and the lower panel is the statistical results. E) Amino acid targeting metabolomics shows the effect of CPS1 on amino acids content in HCC cells. F) Detection of Asp content in shCPS1, CPS1‐OE and NC HCC cells. G) CCK8 assay on the effect of Asp on HCC proliferation. H, I) Transwell detection of Asp regulates HCC invasion. J, K) Wound healing experiments exbibit regulation of HCC cell migration by treated of different concentrations of Asp (0, 200 mM, 1 mM, 5 mM) for 24 h. Scale bar: 200 µm. Values represent the means±SDs. *: *p* < 0.05, **: *p* < 0.01, ***: *p* < 0.001. The above in vitro experiments have all been biologically replicated (n = 3).

Transcriptome sequencing showed that when CPS1 expression was downregulated, the expression of SLC1A3 was reduced subsequently (**Figure** **3**A). Our results exhibited SLC1A3 was positively regulated by CPS1 at both mRNA and protein levels in HCC cells (Figure 3B,C, Figure S4A–C, Supporting Information). Knocking down SLC1A3 increased HCC cell migration (Figure 3D). It is known that SLC1A3 is the main transporter of Asp and glutamine (Gln), and has the function of supporting cell proliferation,^[^ ^28^ ^]^ therefore, we performed amino acid targeted metabolomic assay. There were various amino acids decreased in CPS1‐silenced cells (Figure 3E). As expected, Gln and Asp were dramatically reduced. It is interesting among the detected amino acids, only Asp was directly related to the urea cycle (Cit enters the cytoplasm after formatted in mitochondrial, which will be catalyzed by arginine succinate synthase, and condensed with Asp to synthesize arginine succinate). Intracellular Asp level decreased in shCPS1 cells and increased in CPS1‐OE cells (Figure 3F). Adding exogenous Asp effectively promoted proliferation and inhibited invasion or migration in HCC (Figure 3G–K). We also collected serum samples from HCC patients and healthy individuals to measure Asp levels. Although there was no statistical difference, Asp levels in HCC patients (average value was 0.9683 nmol µL^−1^) were slightly lower than those in healthy individuals (average value was 1.053 nmol µL^−1^) (Figure S4D, Supporting Information). We speculated that as an important amino acid, such a slight difference may be enough to create a butterfly effect, causing cells to produce a series of response reactions, thereby affecting the development of liver cancer. In short, these results demonstrated CPS1 regulates HCC progression through increasing intracellular accumulation of Asp and this process is mediated by SLC1A3.

### 2.5 CPS1 Increased m6A Modification to Stabilize SLC1A3 mRNA

**Figure 4:**
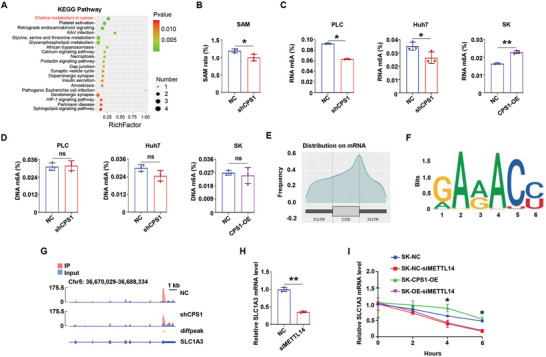
CPS1 increases m6A modification to stabilize SLC1A3 mRNA. A) KEGG analysis of the effect of CPS1 on cellular pathways. B) Detection of S‐adenosylmethionine (SAM) levels in PLC‐NC and PLC‐shCPS1 cells. C, D) Modification of RNA m6A and DNA m6A by CPS1 in PLC, Huh7 and SK cells. E–G) m6A sequencing reveals m6A modification in SLC1A3 mRNA. H) qPCR tests the expression regulation of METTL14 on mRNA levels of SLC1A3. I) Changes in the stability of SLC1A3 mRNA after reduced expression of METTL14 detected by qPCR (actinomycin D 5 µg mL^−1^, separately treated for 0, 2, 4, and 6 h). Values represent the means±SDs. *: *p* < 0.05, **: *p* < 0.01, ns: no significant difference. The above in vitro experiments have all been biologically replicated (n = 3).

CPS1 is a metabolic enzyme, so the non‐targeted metabolomics was used to analyze changes in metabolism between NC group and shCPS1 group. It is noteworthy that choline metabolism was the most prominent (**Figure** **4**A). Choline is known to be a major methyl donor, and higher serum choline levels were associated with better HCC survival.^[^ ^29^ ^]^ In addition, amino acid targeted metabolomic assay revealed that, Methionine (Met) content decreased sharply with CPS1 knockdown (Figure 3E). Met is derived from S‐adenosylmethionine (SAM), which serves as a donor for methyl groups. The above results suggested that CPS1 may regulate methylation modification in HCC. Consequently, we found SMA was reduced in the shCPS1 group (Figure 4B). We examined the regulation of CPS1 on m6A at both RNA and DNA level. Compared with the NC cells, RNA m6A decreased by about 20% in shCPS1 cells, but increased by about 10% in CPS1‐OE cells (Figure 4C). In contrast, the m6A level of DNA did not change significantly (Figure 4D).

We inferred that CPS1 may affect SLC1A3 expression by regulating its RNA m6A modification. The m6A sequencing showed that there was an m6A peak in the 3′UTR region of SLC1A3 (Figure 4E,F). Knockdown of CPS1 reduced the m6A modification of SLC1A3 mRNA (Figure 4G). We then attempt to determine what mediates the m6A modification of SLC1A3 by CPS1. We down‐regulated m6A core writers (METTL3, METTL14, WTAP) separately (Figure S5A–C, Supporting Information) and found that silencing METTL14 decreased the mRNA content of SLC1A3 by half (Figure 4H). The stability of SLC1A3 mRNA was significantly weakened when METTL14 expression was reduced (Figure 4I), while the other writers did not affect the expression of SLC1A3 (Figure S5D,E, Supporting Information). Taken together, we demonstrated that CPS1 upregulated SLC1A3 expression through m6A modification mediated by METTL14.

### 2.6 Loss of CPS1 Activated PC‐PLC/DAG/PKC Axis in HCC Cells

**Figure 5:**
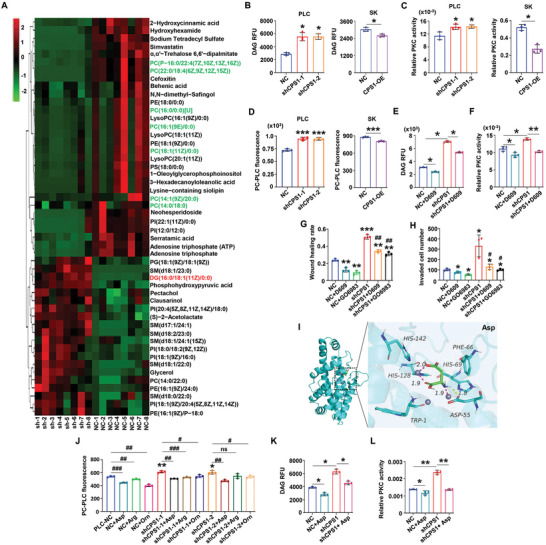
Loss of CPS1 decreases Aspartate to activate PC‐PLC/DAG/PKC pathway in HCC cells. A) Metabolomics detection of changes in metabolites of PLC‐NC and PLC‐shCPS1 cells (n = 8). B–D) Changes in DAG content (B), PKC activity (C), PC‐PLC activity (D) after knocking down or overexpressing CPS1 compared to the control group. E, F) Changes in DAG content and PKC activity in PLC‐NC/shCPS1 cells treated with PC‐PLC inhibitor D609 (10 µM, treated for 24 h). G, H) Effects of PC‐PLC inhibitor D609 and PKC inhibitor GO6983 on migration and invasion of HCC cells (GO6983 5 µM, treated for 24 h). I) Molecular docking analysis of the binding between PC‐PLC and Asp. J) Effects of Asp (200 µM), Arg (200 µM), and Orn (500 µM) on the PC‐PLC activity in HCC cells. All the above amino acids were treated for 24 h. K, L) The regulatory effect of Asp on DAG levels and PKC activity in HCC cells. Values represent the means±SDs. *versus NC, # versus shCPS1, */#: *p* < 0.05, **/##: *p* < 0.01, ***/###: *p* < 0.001. The above in vitro experiments have all been biologically replicated (n = 3).

The non‐targeted metabolomics showed that DAG level increased markedly after knocking down CPS1 level (**Figure** **5**A). DAG is the second messenger of intracellular signal transduction.^[^ ^30^ ^]^ It activates a variety of cancer promoting factors by binding to and activating PKC cascade, and regulates cell proliferation, differentiation, and metastasis.^[^ ^31^ ^]^ We detected the content of DAG after changing the expression of CPS1. Compared with the NC cells, DAG content in shCPS1 cells increased by about 30% and PKC activity increased by 20% approximately, but DAG level was markedly decreased in CPS1‐OE cells, and PKC activity was reduced by half compared to the NC cells (Figure 5B,C). It was illustrated that CPS1 could suppress the DAG/PKC axis in HCC.

The metabonomic results also showed that phosphatidylcholine (PC), which could be converted into DAG, decreased significantly when CPS1 was knocked down (Figure 5A). The PC pathway can be divided into two ways: 1) PC is hydrolyzed to DAG and choline phosphate that catalyzed by PC‐PLC,^[^ ^32^ ^]^ 2) PC forms phosphatic acid by phospholipase D (PLD), and then transform to DAG through the catalysis of phosphatic acid phosphatase.^[^ ^33^ ^]^ Subsequently, we detected the activity of key enzymes in the mentioned above, and found PC‐PLC activity was regulated by CPS1 (Figure 5D, Figure S6A, Supporting Information), while PLD and sphingomyelin synthetase (SMS) did not change significantly (Figure S6B,C, Supporting Information). However, phosphatidylinositol specific phospholipase C (PI‐PLC) activity was slightly affected by CPS1 expression (Figure S6D, Supporting Information). Further analysis of metabolomics revealed that urea cycle, phospholipid metabolism, and protein kinase were significantly influenced by CPS1 (Figure S6E, Supporting Information). We found D609, a main PC‐PLC inhibitor, could reduce the content of DAG in HCC cells (Figure 5E) and the activity of PKC (Figure 5F). In addition, the migration and invasion of tumor cells were also inhibited after D609 treatment, which effect was similar with the PKC inhibitor GO6983 (Figure 5G,H, Figure S6F,G, Supporting Information). It could be seen from these results that downregulation of CPS1 facilitates the transformation of PC into DAG by enhancing the PC‐PLC activity, thus promoting PKC, and finally strengthening ability of migration and invasion in HCC cells.

### 2.7 Loss of CPS1 Activated PC‐PLC/DAG/PKC Axis by Decreasing Asp in HCC Cells

Tumor metabolism reprogramming is a complex system where metabolites regulate the expression of genes and proteins.^[^ ^34^, ^35^ ^]^ As Asp was one of the top amino acids reduced in CPS1‐knockdown cells and it was involved in urea cycle, we supposed that Asp might have a regulatory effect on PC‐PLC. Through molecular docking analysis, we found there was a binding site of Asp, Arg and Orn (the intermediate metabolites of urea cycle) in the PC‐PLC protein structure (Figure 5I, Figure S6H, Supporting Information). Asp had the strongest binding ability with PC‐PLC, forming hydrogen bond interactions with Asp‐55 at the active site of PC‐PLC (Table S2, Supporting Information). After treated PLC‐NC and PLC‐shCPS1 cells with Asp, Arg and Orn, the PC‐PLC activity weakened accordingly, but only Asp had a stable inhibitory effect on PC‐PLC activity (Figure 5J). Consistently, Asp reduced the abundance of intracellular DAG content (Figure 5K) and inhibited the activity of PKC (Figure 5L). Our results showed that Asp mediated the inhibitory function of CPS1 on HCC migration and invasion through suppressing the PC‐PLC/DAG/PKC axis.

### 2.8 CPS1 Negatively Regulated the Expression of MMP1/CCL5/ALDH1A3 in HCC Cells

**Figure 6:**
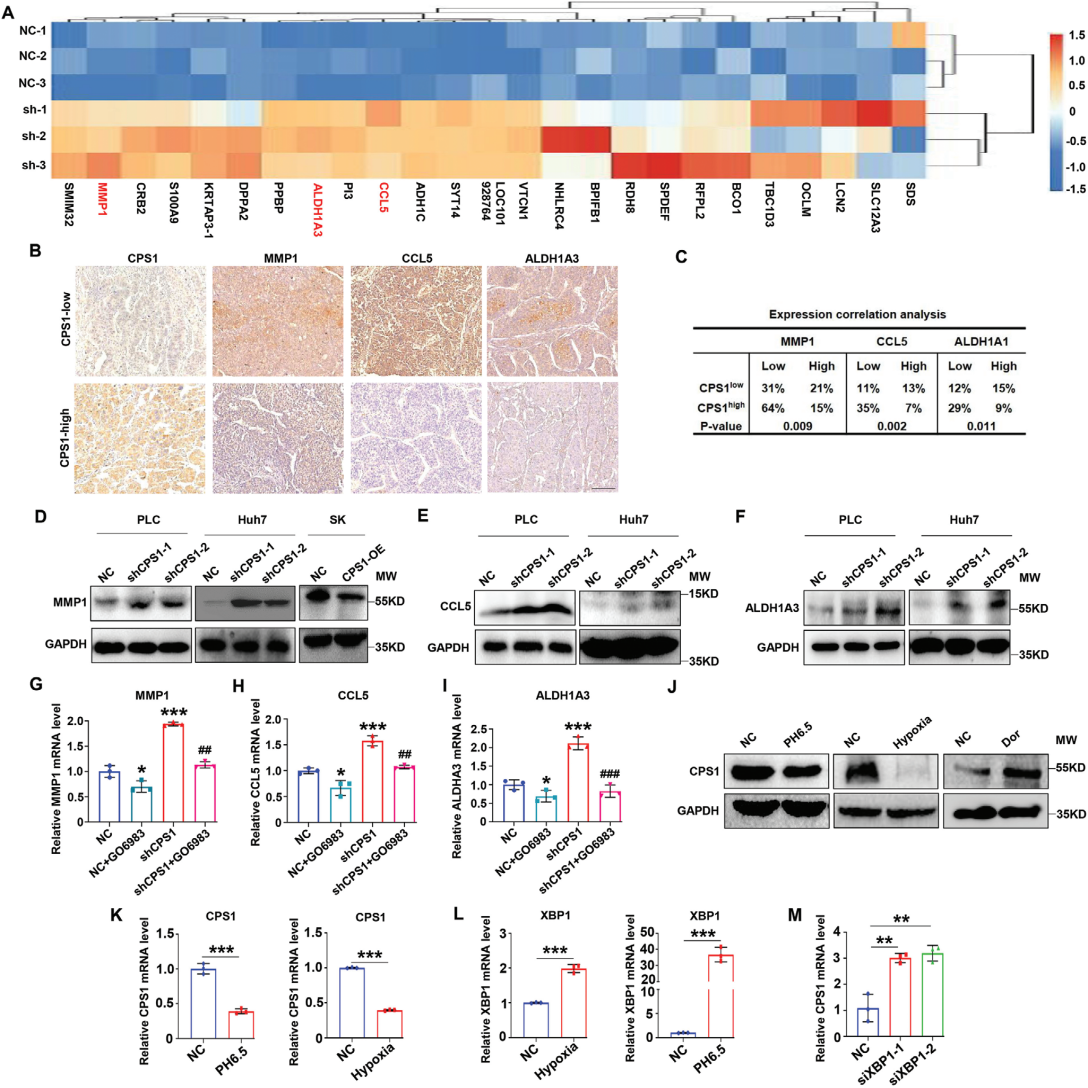
Upstream and downstream regulation of CPS1. A) Transcriptome heatmap analysis of the top 30 genes upregulated in shCPS1. B) IHC analysis of the correlation between CPS1 and MMP1, CCL5, ALDH1A3 in HCC patients (n = 50). C) Correlation analysis statistical chart. D–F) WB detection of the regulatory effect of CPS1 on MMP1 (D), CCL5 (E) and ALDH1A3 (F) expression. G–I) Change in MMP1 (G), CCL5 (H) and ALDH1A3 (I) expression after PKC inhibitor treatment detected by qPCR (GO6983 5 µM, treated for 24 h). J) WB detection of expression of CPS1 after treatment with pH 6.5, hypoxia (1% O_2_), and AMPK inhibitor Dorsomorphin dihydrochloride (Dor, 2 µM), treated for 24 h. K, L) qPCR detection of CPS1 and XBP1expression after treatment with pH 6.5, hypoxia (1% O_2_), and Dor (2 µM), treated for 24 h. M) qPCR detection of CPS1 expression in XBP1‐knockdown PLC cells. Scale bar: 200 µm. Values represent the means±SDs. *: *p* < 0.05, **: *p* < 0.01, ***: *p* < 0.001. The above in vitro experiments have all been biologically replicated (n = 3).

We tried to further reveal the downstream signaling of PKC. Whole transcriptome resequencing helped us to screen out the top three up‐regulated genes: matrix metalloproteinase 1 (MMP1), Chemokine ligand 5 (CCL5) and acetaldehyde dehydrogenase family 1‐A3 (ALDH1A3) in shCPS1 cells (**Figure** **6**A). MMP1 belongs to the matrix metalloproteinase family, which degrades collagen, gelatin and other substrates to promote the development of liver cancer.^[^ ^36^ ^]^ CCL5 and ALDH1A3 have been found to have a pro‐tumorigenic effect in various cancers,^[^ ^37^, ^38^ ^]^ all of them have the function of promoting invasion and metastasis.

Clinical HCC samples also showed a negative correlation between the expression of CPS1 and MMP1/CCL5/ALDH1A3 (Figure 6B,C). Obviously, CPS1 negatively regulated the expression of MMP1/CCL5/ALDH1A3 at both mRNA and protein levels (Figure 6D–F, Figure S7A–F, Supporting Information). After treating HCC cells with PKC inhibitor GO6983, expression of MMP1, CCL5 and ALDH1A3 were decreased (Figure 6G–I). It was noted that m6A sequencing did not detect MMP1, CCL5, and ALDH1A3, indicating these genes are not regulated by m6A. Overall, we revealed that CPS1‐induced Asp could inhibit the PC‐PLC/DAG/PKC axis to downregulate MMP1, CCL5 and ALDH1A3, thus depressing HCC invasion and metastasis.

### 2.9 Environmental Pressure Could Inhibit the Expression of CPS1

The occurrence and development of tumors are often accompanied by changes in the internal and external microenvironment. Acidic microenvironment and hypoxia are two common phenomena in malignancy.^[^ ^39^, ^40^ ^]^ We found acidic microenvironment (PH6.5) and hypoxia lead to a decrease in CPS1 expression, but AMPK inhibitor (Dorsomorphin dihydrochloride) increased CPS1 protein and mRNA level (Figure 6J,K). In the two adverse microenvironments mentioned above, X‐Box Binding Protein 1 (XBP1), an important endoplasmic reticulum (ER) stress gene,^[^ ^41^ ^]^ was markedly elevated (Figure 6L). Furthermore, downregulation of XBP1 led to an increase in CPS1 mRNA level (Figure 6M, Figure S7H, Supporting Information). These results hinted that the unfavorable environment may regulate the expression of CPS1 through ER stress.

### 2.10 The Prospect of CPS1 in the Treatment of HCC

**Figure 7:**
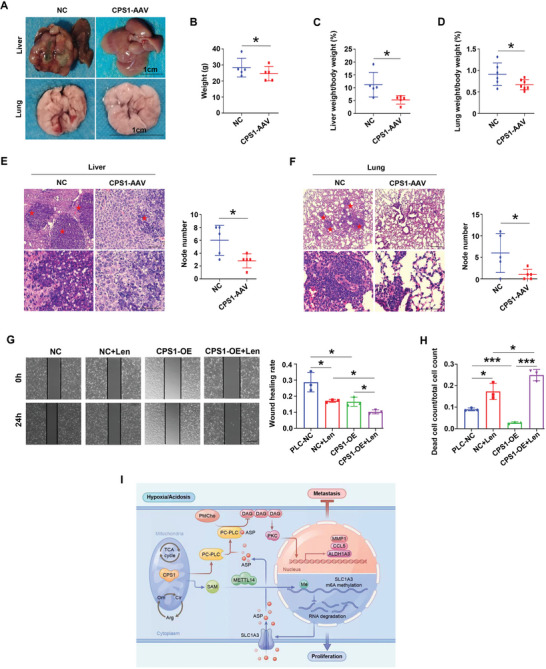
Exploration of the therapeutic prospects of CPS1 for HCC. A) Comparison of liver and lung tumor status between CPS1‐AAV treatment group and untreated group in mice with spontaneous liver cancer, *n* = 5. B–D) Statistics of body weight (B), liver weight ratio (C), and lung weight ratio (D), of CPS1‐AAV treatment group and untreated group. E, F) HE staining shows liver and lung tumor nodules. The red five‐pointed star indicates tumor area. G) Wound healing experiment tests the effect of CPS1 on the efficacy of Lenvatinib (Len, 10 µM, treated for 0 and 24 h). The left panels are wound healing images, the right panel is a statistical chart of wound healing rate. H) Mortality statistics of NC cells and CPS1‐OE cells in response to Len effect (Len, 10 µM, treated for 0 and 48 h). I) Schematic diagram of the mechanism by which CPS1 regulates HCC. Scale bar: 200 × magnification, 100 µm, 100 × magnification, 200 µm. Values represent the means±SDs. *: *p* < 0.05, **: *p* < 0.01, ***: *p* <0.001. The above in vitro experiments have all been biologically replicated (n = 3).

Since CPS1 has such an important anti‐cancer effect, we want to know if this gene has clinical application value. We injected CPS1‐AAV into three‐weeks old mice (which has the ability to spontaneously form HCC) via tail vein. Four weeks later, we were pleasantly surprised to find that CPS1‐AAV had the ability to inhibit liver cancer progression and distant lung metastasis (**Figure** **7**A). In addition, the CPS1‐AAV group showed a significant decrease in body weight, liver weight ratio, and lung weight ratio (Figure 7B–D). Figure 7E,F showed that, compared with the control group, CPS1‐AAV treatment group had fewer tumors and lung metastases. Based on CPS1's anti‐tumor effect, we hope it can improve the efficacy of liver cancer drugs. Our results displayed, the inhibitory effect of CPS1‐overexpression combined with Lenvatinib on HCC cell migration was significantly better than Lenvatinib monotherapy (Figure 7G). Moreover, The cell death rate sharply elevated in combination therapy group as well (Figure 7H). Thus, CPS1 might be an efficient target for HCC treatment.

In summary, CPS1 regulates HCC progression through the SLC1A3‐Asp‐PC‐PLC‐DAG/PKC pathway (Figure 7I).

## 3 Discussion

The urea cycle is an important ammonia metabolism pathway in hepatocytes, which plays a role not only in preventing ammonia poisoning, but also connects the TCA cycle and is closely related to glucose metabolism, lipid metabolism, and amino acid metabolism.^[^ ^42^ ^]^ We found in this study that CPS1, the rate‐limiting enzyme of the urea cycle, was significantly reduced or even absent in HCC tissues. CPS1 expression was associated with less macrovascular invasion and favorable prognosis. CPS1 suppressed invasion and metastasis, but facilitated the m6A modification of Asp transporter SLC1A3 to fuel cell proliferation, the mechanism may be CPS1‐downregulation decreased the intracellular Asp level to activate the PC‐PLC/DAG/PKC axis. We also found that the hostile tumor microenvironment (TME), such as hypoxia, acidosis and nutrition deprivation, could cause the downregulation of CPS1, which might be mediated by the ER stress response. These findings reflect the dynamic demand for Asp during cancer progression. We propose that CPS1 is important for HCC cell proliferation when there are adequate nutrients, but it is deregulated to facilitate the invasion and metastasis of HCC cells when the TME becomes hostile. This finding is consistent with that of Matteo Rossi, who reported that the loss of phosphoglycerate dehydrogenase (PHGDH) potentiates metastatic dissemination of breast cancer cells, although the catalytic activity of PHGDH supports cancer cell proliferation.^[^ ^43^ ^]^

Our results indicated that CPS1 decreased DAG level by regulating the activity of PC‐PLC. PL‐PLC belongs to the Phospholipase C family and is the main enzyme in the photosphatidylcholine cycle.^[^ ^45^ ^]^ It can hydrolyze glycophorophospholipid to produce DAG and photosphaline, and participate in various physiological or pathological processes such as cell proliferation, differentiation, and apoptosis.^[^ ^45^, ^46^, ^47^ ^]^ Lorio et al. found that the activity of PC‐PLC increases in ovarian cancer, and promotes cancer cell proliferation.^[^ ^45^ ^]^ PC‐PLC also promotes invasion and migration of breast cancer,^[^ ^46^ ^]^ which is related to the regulation of peripheral growth factor receptor‐2.^[^ ^47^ ^]^ In addition, downstream lipid products of PC‐PLC can activate mitogen activated protein kinase (MAPK) and NF‐κB signaling, affecting the development of leukemia.^[^ ^48^ ^]^ Limited research on the relationship between PC‐PLC and HCC, a study reports that PC‐PLC can promote proliferation of rat liver cancer cells^[^ ^49^ ^]^ however, the regulation of PC‐PLC on progression of liver cancer is unclear. Our study demonstrated that CPS1‐induced Asp accumulation can inhibit the activity of PC‐PLC. Molecular docking analysis revealed that Asp could bind to yeast PC‐PLC protein, implying that Asp could directly inhibit the activity of PC‐PLC. Similarly, a latest study demonstrated that arginine could bind with RNA‐binding protein RBM39 to promote liver cancer formation.^[^ ^50^ ^]^ Unfortunately, the protein structure of human PC‐PLC has not been elucidated, only its activity can be detected currently. This is an unresolved issue that requires further investigation. Furthermore, we found that MMP1/CCL5/ ALDH1A3, which have been identified as cancer‐promoting genes,^[^ ^36^, ^37^, ^38^, ^51^, ^52^ ^]^ were the downstream targets of the CPS1/PC‐PLC/DAG/PKC axis.

CPS1 elevated the Asp level seems contradictory to the urea cycle process as urea cycle consumes Asp. The sources of Asp in cells come from: 1) intracellular synthesis, which derives from the conversion of glucose. Glucose can be ultimately generated α‐ ketoglutaric acid through TCA cycle. α‐ketoglutarate undergoes transaminase reaction with glutamic acid to produce Asp and glutamine. Glucose also can be converted into phosphopyruvate, which then is converted to aspartate phosphate by glyoxylate synthase. Aspartic acid phosphate undergoes the catalysis of aspartic acid phosphotransferase to remove a phosphate group and generate Asp. 2) Extracellular uptake by transporters. 3) The conversion from other amino acids, such as glutamic acid, which undergoes transamination with pyruvate to produce Asp and glutamine. In this study, metabolomics analysis did not detect changes in both α‐ketoglutarate and phosphopyruvate, thus the glucose synthesis pathway was excluded. After knocking down CPS1, glutamic acid was not changed significantly, and the content of conversion product glutamine was decreased. Therefore, the source of Asp was not in line with amino acid mutual conversion. Based on the above, Asp may mainly come from extracellular uptake.

Transcriptome sequencing showed that after knockdown of CPS1 expression, the main Asp transporter SLC1A3^[^ ^53^ ^]^ level was decreased. Sun et al. demonstrated that loss of SLC1A3 function resulted in an approximately 8‐fold decrease in intracellular Asp and a 1.5‐fold decrease in glutamine in PC3 cells.^[^ ^54^ ^]^ This should be the main reason for the decrease in Asp content.

SLC1A3 is involved in various processes of tumor development, but research on it mainly focuses on regulating tumor cell proliferation.^[^ ^53^, ^54^, ^55^ ^]^ There is a lack of research on the relationship between SLC1A3 and liver cancer metastasis. Similar to SLC1A3, research on Asp promoting cell proliferation is dominant,^[^ ^22^, ^23^, ^24^ ^]^ and few studies have found that Asp enhances tumor cell metastasis.^[^ ^25^, ^26^ ^]^ Our results revealed that Asp has the doubled‐edged sword function, promoting HCC cell proliferation while inhibiting invasion. We further revealed that CPS1 could increase the intracellular SAM level, the main donor for methylation modification. We found that CPS1 significantly enhanced the SLC1A3 RNA m6A modification to increase its stability. As is known that m6A modification needs writers, readers and erasers. We revealed that CPS1‐induced SLC1A3 m6A modification is mainly by the writer METLL14. It has been reported METLL14 mediates N‐methyladenosine modification to induce Developmentally‐Downregulated 4 upregulation to promote HCC growth and metastasis.^[^ ^56^ ^]^

## 4 Conclusion

We demonstrated that negative CPS1 expression in HCC tissues or CTCs correlated with advanced tumor stage. Loss of CPS1 inhibits HCC proliferation, but facilitates the metastasis of HCC. In the molecular mechanism, loss of CPS1 could reduce the SAM level to decrease m6A modification of SLC1A3 mainly mediated by METTL14, causing SLC1A3 mRNA instability and less Asp import to the cell. Asp fuels cell proliferation and meanwhile inhibits the PC‐PLC/DAG/PKC axis.

## 5 Experimental Section

### Cell Lines and Cell Culture

Human liver cancer cell lines Huh7, PLC, HepG2, SK, were obtained from the American Type Culture Collection (Manassas, VA, USA), and 7721 was bought from Beyotime Ltd. (Shanghai, China). All cells were identified by short‐tandem‐repeat profiling to ensure no mycoplasma contamination. Cells were cultured in Dulbecco's Modified Eagle Medium (DMEM) pH 7.4 (HyClone, USA) supplemented with 10% fetal bovine serum (Gibco, USA), 100 units mL^−1^ penicillin, and 100 µg mL^−1^ streptomycin (HyClone). Cells were cultured in a 37 °C humidified atmosphere containing 5% CO_2_.

### Clinical Samples

All clinical tissues were collected from 2013 to 2018 at the Second Affiliated Hospital of Chongqing Medical University after informed consent was obtained from all patients. The patients received no chemotherapy or radiotherapy before surgery. These clinical samples were stored at the Department of Pathology. The diagnosis of HCC was estimated independently by at least two histopathologists. The stage of HCC was decided according to the BCLC stage system, which divide HCC into BCLC 0/A/B/C/D stages. This study was carried out according to the principles of the Helsinki Declaration and approved by the Ethical Committee of the Second Affiliated Hospital of Chongqing Medical University ((2022) 411). Eight cases of fresh HCC and adjacent tissues were used for proteomic analysis, 152 cases paraffin‐embedded tissues were used for CPS1 IHC staining, 10 cases frozen tissues were used for qPCR assay.

### CTC Acquisition

The CTCs were detected in 4 mL peripheral blood from 34 initially‐diagnosed HCC patients by CTC‐100, which was a microfluidic‐based platform (Cellomics, China). This method enriches CTCs according to the cell size and was label‐free utilizing the inertial focusing principle. The enriched cells were validated via immunostaining with EpCAM antibody, CD45 antibody, and DAPI. The characteristics of CTCs were CD45 negative and DAPI positive, and the nucleus was larger than 15µm.^[^ ^57^ ^]^

### Lentivirus or Adenovirus Infection

Lentiviruses carrying small hairpin RNA (shRNA) sequence of human CPS1 were used to decrease CPS1 expression, while adenovirus containing whole CDS of CPS1 gene (CPS1‐AAV) was used for overexpression experiment. All viruses were purchased from Obio Company (Shanghai, China). Cells were cultured in 24‐well plates at 1 × 10^5^ cells/well, and lentivirus or adenovirus was added into the medium separately (MOI = 20). After three days, puromycin was added to screen the stable infected cells.

### siRNA Interference Experiment

When the fusion rate of cells reached 60% in the 6‐well plates, the culture medium was replaced with Opti‐MEM (Gibco, USA). Lipofectamine 3000 (Thermo Fisher, USA) and siRNA were well mixed before added into culture medium. After 8 h, replaced the culture medium with complete DMEM. Please refer to the user manual (Cat. No. L3000001) for specific instructions.

All oligonucleotide sequences used in this study were shown in Table S1 (Supporting Information).

### Proteomic Analysis

The proteomic analysis was completed by Oebiotech (Shanghai, China). In short, HCC tissues were taken out for total protein extraction. The total protein was digested and cut into polypeptide fragments, and then separated by HPLC (EASY‐nLC TM 1200 liquid‐phase system, Thermo, USA). The total protein also was fractionated into Mass Spectrometry (MS)(Thermo Q‐Exactive mass spectrometer, Thermo, USA)electric field for further ionization. MS obtained the information of mass charge ratio and peak type of each ion. Then the software (Proteome Discoverer TM 2.2, Thermo, USA) was used to calculate the amino acid composition. Finally, the qualitative and sequence information of the protein were obtained by searching and comparing the database (Uniprot human database and Hepatitis B virus database).

### 3D Invasion Detection

The vertical invasion model was carried out according to this previous work.^[^ ^58^ ^]^ About 1 × 10^4^ cells were added into 96‐well plates in triplicate and allowed 24 h for complete cell adhesion. Then removed the medium and added 50 µL 50% Matrigel (Corning, USA) and 50% Collagen I mixture (Thermo Fisher, USA) to cells and cultured for 48 h to allow cells to vertically invade. Layers were scanned by using a high content instrument (ImageXpress Micro, Molecular Device, USA) for a total of 100 µm with 10 µm interval. Finally, cell numbers were counted for each layer by a high content analysis system (Image‐Pro Plus 6.0) to compare the invasion ability between different sample groups.

### Wound Healing Assay

When cell fusion rate reached more than 90% in the 6‐well plates, a straight line was drawn in the cells by a 10 µL pipette tip along the sterilized ruler. Cells were washed with phosphate buffer (PBS) for 3 times. Cell migration was observed and recorded at 0 h and 24 h.

### Cell Migration and Invasion Assays

Twenty‐four‐well transwell chambers were used for this assay (BD Falcon, NJ). Briefly, 3 × 10^4^ cells were plated into each upper chamber with 8‐µm pores and cultured in 200 µL serum‐free DMEM. The lower chambers were filled with 500 µL complete DMEM. After incubation for 24 h at 37 °C, cells that migrated in the lower chambers were fixed with 4% polyformaldehyde and stained with 0.1% crystal violet. The number of cells was counted in 5 distinct areas at ×100 magnification. The results represent the average cell number in 3 wells per cell line. Then, the upper surface of the polycarbonate filter was coated with 10% Matrigel TM (BD Biosciences, NJ), and 5 × 10^4^ cells were added to detect cell invasion. The other conditions were the same as those in the migration assay.

### Orthotopic Liver Tumor Model

Briefly, server combined immune‐deficiency (SCID) mice were anesthetized and liver were exposed after laparotomy. Cells of 1 × 10^6^ of each group in 50 µL DMEM were injected into livers of mice, and then the abdomen was sutured. After 4 weeks, tumor formation was detected by fluorescence imaging (AniView Model animal live imaging system, Photo Technology, China). Livers and lungs were collected for pathological test when all mice were sacrificed. The tissues were dissected into 5 mm thick pieces and each piece was subjected to dehydration, and paraffin embedding. Pathological section with 4 µm thickness were made and stained with HE. The metastatic foci in liver and lung tissues were identified by a pathologist.

### Nude Mouse Subcutaneous Tumor Model

Four‐week‐old male nude mice were purchased from Byrness Weil biotech Ltd (Chongqing, China). Growing cells of 1 × 10^7^ in 100 µL PBS were injected into the axilla of each mouse. The tumor size was measured every week, and the tumors were collected four weeks later.

### Spontaneous Tumor Formation Mouse Model

Spontaneous tumor forming mice were purchased from Cyagen company (Cyagen Biosciences, USA), with the genotype Alb Cre+/MYC+. When the mice were three weeks old, 100 µL physiological saline containing CPS1‐AAV (1 × 10^8^ PFU/mL/mouse) was injected through the tail vein. The control group mice were only injected with 100 µL physiological saline. CPS1‐AAV treated and untreated mice were fed in the same environment. All mice were sacrificed after four weeks.

All mouse experiments were carried out according to the principles of the Helsinki Declaration and approved by the Ethical Committee of the Second Affiliated Hospital of Chongqing Medical University (Approval No. 2021–115).

### Non‐Targeted Metabonomic Analysis

The metabonomic analysis was completed by Oebiotech (Shanghai, China). Briefly, 5 × 10^7^ cells were processed using ultrasonic homogenizer in a mixture of methanol and water (1/4, vol/vol). L‐2‐chlorophenylalanine (0.3 mg mL^−1^) dissolved in methanol was used as the internal standard. The dried supernatant was then dissolved in pyridine containing 15 mg mL^−1^ methoxyamine hydrochloride. The samples were then subjected to LC‐MS and GC‐MS analysis. The obtained GC/MS raw data in .D format were transferred to .abf format via software Analysis Base File Converter for quick data retrieval. Then, data were imported into software MS‐DIAL, which performs peak detection, peak identification, MS2Dec deconvolution, characterization, peak alignment, wave filtering, and missing value interpolation. Metabolite characterization was based on LUG database. The original LC‐MS data were processed by the Progenesis QI V2.3 software (Nonlinear, Dynamics, Newcastle, UK) for baseline filtering, peak identification, integral, retention time correction, peak alignment, and normalization. Main parameters of 5 ppm precursor tolerance, 10 ppm product tolerance, and 5% product ion threshold were applied. Compound identification was based on precise mass‐to‐charge ratio (M/z), secondary fragments, and isotopic distribution using The Human Metabolome Database (HMDB), Lipidmaps (V2.3), Metlin, EMDB, PMDB, and self‐built databases to do qualitative analysis. Variable Importance of Projection (VIP) values obtained from the OPLS‐DA model were used to rank the overall contribution of each variable to group discrimination. A two‐tailed Student's t‐test was used to verify whether the difference of metabolites between groups were significant. Differential metabolites were selected with VIP values greater than 1.0 and p‐values less than 0.05.

### Targeted Amino Acid Detection

Targeted amino acid also was detected by Oebiotech (Shanghai, China). LM precise targeting‐amino acid detection: sample processing was the same as non‐targeted metabolomics. Chromatographic conditions: DB‐5MS capillary column (30 m × 0.25 mm × 0.25 µm, Agilent J&W Scientific, Folsom, USA). The carrier gas was high‐purity helium (purity not less than 99.999%), the flow rate was 1.2 mLmin^−1^, and the temperature of the sample inlet was 300 °C. Injection volume was 1 µL. No split injection, solvent delayed for 4 min. Programmed temperature rise: the initial temperature of the column temperature box was 50 °C for 0.5 min, followed by a programmed temperature rise of 15 °C min^−1^ to 125 °C for 2 min, and a programmed temperature rise of 8 °C min^−1^ to 210 °C for 2 min, Raise the temperature to 270 °C at 11 °C min^−1^ for 1 min, and raise the temperature to 305 °C at 25 °C min^−1^ for 3 min.

Mass spectrometry conditions: electron bombardment ion source (EI), with an ion source temperature of 300 °C and a transmission line temperature of 280 °C. The scanning method was selective reaction detection scanning (SRM), with a quality scanning range of m/z: 40–600. Metabolite quantification was analyzed using selective reaction monitoring using triple quadrupole mass spectrometry. In the single ion detection scanning mode, the EI will send gas molecules from the chromatographic column end, which will form charged ions through electron bombardment. Then, specific mother ions will be selected to collide and form daughter ions, which will enter the quadrupole mass analyzer for screening and separation of ions with different mass to charge ratios. They will enter the electron multiplier to generate electrical signals, ultimately obtaining 2D information of each substance and raw data. The metabolite ion fragments were confirmed by spectral library and standard samples. Finally, the ion chromatographic peaks were integrated by peak area, and the chromatographic peaks of the same substance in different samples were manually integrated and corrected to obtain quantitative results.

### Whole Transcriptome Sequencing

The whole transcriptome sequencing was achieved by Oebiotech (Shanghai, China). RNA from CPS1‐knockdown cells and control cells was extracted and reversed to cDNA. After amplification and purification, they were sequenced on the computer after passing the quality inspection (Oebiotech, Shanghai, China). The differentially expressed genes were screened by the following criterion: fold change larger than 2 and p‐value less than 0.05. These genes were applied to enrichment analysis by KEGG (http://www.genome.jp/kegg/) and GO (http://geneontology.org/) database.

### Cell Proliferation Assay

Cells were plated in 96‐well plates (2000 cells/well) in triplicate and measured using Cell Counting Kit‐8 (CCK‐8) (Dojindo Laboratories, Japan). Cell proliferation was determined every 24 h for three days following the manufacturer's protocol. The absorbance at 450 nm was measured with a microplate reader (Bio‐Rad, USA).

### Colony Formation Assay

Long‐term survival of cells was assessed by their ability to form colonies. 1000 cells were seeded in a 6‐well plate per well. After 30 days, colonies were fixed in 4% paraformaldehyde and stained with 0.1% crystal violate (Invitrogen, USA) before counting.

### Cell Apoptosis Assay

Cells were obtained at a density of 1.0 × 10^6^ cells mL^−1^ and washed with PBS, then incubated with reagents from the Annexin‐V‐FITC Apoptosis Detection Kit (Neobioscience, China) according to the manufacturer's protocol. Cells were analyzed by FACS Vantage SE flow cytometer (BD Biosciences, USA).

### Aspartate Detection Assay

Aspartate assay kit Ab102512 (Abcam, UK) was used to detect the content of aspartate. Mixed 10 µL cell extracts or serum, 2 µL Aspartate Enzyme Mix, 2 µL Conversion Mix, 2 µL Probe. Then 44 µL Aspartate buffer were added to each reaction system. The mixture was incubated at room temperature for 30 minutes, and the OD value was measured at 570 nm.

### Phospholipase C Activity Assay

Phospholipase C (PLC) Activity Assay Kit (ab273343) was used to detected PLC activity (Abcam, UK). Reaction mix containing PLC Assay Buffer and PLC Substrate (50 µL), which was added into positive control and sample wells. Background mix (50 µL) was added into negative control wells. They were incubated for 60 minat 37 °C. The OD was measured at 405 nm. The standard curve was used to calculate cell PLC activity.

### PLD Assay

Ab183306 reagent kit (Abcam, UK) was used to detecting PPLD activity. Briefly, 100 µL PLD Assay Buffer was used to clean cell clusters (5 × 10^6^ cells), and the supernatant was collected. The reaction mix contained PLD Assay Buffer, PLD Enzyme Mix, PLD Probe and PLD Substrate. Add 50 µL Reaction Mix into standard, sample and positive control wells, then add 50 µL Background Reaction Mix to negative control samples. Measure output at OD570 nm in the dark.

### SMS Assay

SMS activity was carried out according to the instructions in ab138876 (Abcam, UK). Briefly, 50 µL assay mixture was added into each well of SMS standards, blank control and test samples (the total volume of each well was 150 µL), and incubated at room temperature in the dark. The absorbance at 655 nm was measured 1–2 h later.

### DNA m6A Detection

The experiment was conducted according to the instructions of Ab233488 (Abcam, UK). 100–300 ng DNA was extracted and ensured 260/280 > 1.8. Predetermine the number of strip‐wells, 80 µL Binding Solution was added to each well. Then 2 µL Negative control, 2 µL Positive control, and 1–8 µL samples were added into the appropriate wells, mix gently. After incubating at 37 °C for 90 min, wash each well for two times. Antibody (50 µL) was added to each well, incubating for 60 min at room temperature. After being cleaned again, Diluted Detection Antibody (50 µL) and Diluted Enhancer Solution (50 µL) were added in wells separately, and clean them in sequence. After the last cleaning, treating with 100 µL Detection Solution for 1–10 min. 100 µL Stop Solution could end the reaction. Read the absorbance at 450 nm.

### RNA m6A Detection

RNA m6A detection was performed according to the instruction of Ab85912 (Abcam, UK). The steps were similar with that of DNA m6A detection.

### m6A Sequencing Detection

First, total RNAs were extracted and Oligo‐dT magnetic beads were used to enrich the mRNA containing polyA in total RNA. Next, the complete mRNA will be fragmented, and divided into two parts. One part was enriched with m6A methylated mRNA fragments by adding antibodies that can capture m6A, the other part was used as input to directly construct a conventional transcriptome sequencing library. Enrichment of m6A antibodies using magnetic beads, recovery of mRNA fragments containing m6A, and construction of a conventional sequencing library following the transcriptome construction process. Perform high‐throughput sequencing on the constructed sequencing libraries by Illumina Hiseq X Ten (Illumina, USA). After quality control, comparing it with the reference genome. Finally, RIP quality inspection was conducted to evaluate the methylation enrichment efficiency. Methylation peaks and positions were detected by reference genome comparison results, that helped to perform subsequent methylation peak correlation analysis. All software and databases for this project were as follows: fastp v0.20.0, Me TDiff 1.1.0, hisat2 2.1.0, Guitar 1.1.1.18, ChIPseeker 1.12.1, htseq‐count 0.9.1, cufflinks 2.2.1, DESeq2 1.14.1, CIRI 2, Genome (http://ftp.ncbi.nlm.nih.gov/genomes/all/GCF/000/001/405/GCF_000001405.39_GRCh38.p13/GCF_000001405.39_GRCh38.p13_genomic.fna.gz), Gff (http://ftp.ncbi.nlm.nih.gov/genomes/all/GCF/000/001/405/GCF_000001405.39_GRCh38.p13/GCF_000001405.39_GRCh38.p13_genomic.gff.gz), GO and KEGG.

### RNA Stability Experiment

When the cells reached 70–80% confluence, 5 µg mL^−1^ ActD(MedChemExpress, USA) was added and RNA was extracted at 0 h, 2 h, 4 h, and 6 h. Then the expression level of the target genes was analyzed by qPCR. The RNA half‐life was calculated, and GAPDH was used as the internal reference.

### Molecular Docking

PubChem database(https://pubchem.ncbi.nlm.nih.gov/)was used to analyze compounds. Chem3D software helped to optimize and minimize energy using the MM2 module. Ligand molecules were saved as sdf format file for molecular docking. Afterwards, import Maestro 121 212.8 software and optimize it using the LigPrep module, with the force field selected as OPLS3e. The protein structure of PC‐PLC (PDB ID: 2HUC) was derived from the RCSB database(https://www.rcsb.org/).The protein structure was processed on the Maestro 11.9 platform to remove structurally unreasonable structures. The protein was treated with Schrodinger's Protein Preparation Wizard to minimize energy and optimize geometric structure. The processing and optimization of molecular docking was completed by the Glide module in Schr ö dinger Maestro software. Protein processing utilizes the Protein Preparation Wizard module. Preprocess, optimize, and minimize the receptor (using OPLS3e force field for constraint minimization). The compound structure was prepared according to the default settings of the LigPrep module. When screening in the Glide module, the prepared receptor was imported, and its binding site was determined based on the original ligand of the protein structure and prediction (Binding Site Detection). The box size was set to 10 Å x 10 Å x 10 Å. Finally, molecular docking and screening were performed using the Standard Precision docking (SP) method. Analyze the interaction patterns between compounds and target proteins, and obtain information on the interactions between compounds and protein residues, such as hydrogen bonding, π – π interactions, electrostatic interactions, hydrophobic interactions, etc. Based on the docking scores of compounds, it was speculated whether the screened compounds have certain activity.

### Statistical Analysis

All values were presented as means ± standard deviation. All data were obtained from at least three repetitions of each experiment. Prism 8.0 (GraphPad, USA) software was used to analyze the data. All data were shown as mean ± SD. Student's t‐test was used to analyze differences between two groups. The Kaplan‐Meier curve was applied to analyze the survival time between groups. One‐way ANOVA was used to compare three or more groups. Probability values less than 0.05 was considered statistically significant.

### Ethics Approval Statement

This study was carried out according to the principles of the Helsinki Declaration and approved by the Ethical Committee of the Second Affiliated Hospital of Chongqing Medical University ((2022) 411).

## Conflict of Interest

The authors declare no conflict of interest.

## Author Contributions

S.H., M.Y., Z.C., and Z.Z. studied concept, experimental design and performed supervision. Z.Z. and S.C. performed data interpretation, drafting, revision and finalization of the manuscript and funding application. S.C., Q,T., M.H., S.S., X.W., Y.Z. performed data acquisition, analyze, and manuscript drafting. Z.Y., S.L., L.Z., Q.W., H.L., W.Z. performed data acquisition and analysis. All authors have read and approved the manuscript for publication.

## Supporting information



Supporting Information

## Data Availability

The data that support the findings of this study are available in the supplementary material of this article.
