# Supplementary material for: Loss of Carbamoyl Phosphate Synthetase 1 Potentiates Hepatocellular Carcinoma Metastasis by Reducing Aspartate Level
Source: Adv Sci (Weinh). 2024 Oct 10;11(45):2402703. doi: 10.1002/advs.202402703 (PMC11615744; doi:10.1002/advs.202402703)
Supplement: Supplementary file 1 — Supporting Information [file ADVS-11-2402703-s001.docx]

**Supplementary materials and methods**

**RNA extraction and qPCR**

Total RNA was extracted from each sample with TRIzol Reagent (Takara, Japan). The cDNA was obtained by using the PrimeScript RT Master Mix (Takara, Japan). About 50ng of each cDNA was amplified as a template, and qPCR was performed in a CFX96 real-time PCR system (Bio-Rad, USA) with SYBR^®^ Premix Ex TaqTM II kit (Takara, Japan). The PCR primers used are listed in supplementary Table 1.

**Western blotting**

Whole-cell lysates were extracted in a lysis buffer (containing PMSF) (Beyotime, China) and were separated by 10% or 12% SDS-PAGE, then transferred to polyvinylidene fluoride membranes (Millipore, USA) by using a wet transfer apparatus (Bio-Rad, German). Blots were blocked in 5% (w/v) skim milk for 2 h at room temperature and then incubated with antibodies against CPS1, MMP1, SCL1A3 (Abcam, UK), CCL5, ALDH1A3 (Proteintech, China) and GAPDH (ZSGB-BIO, China) overnight at 4 °C. The HRP-coupled anti-rabbit or anti-mouse secondary antibodies were used to interact with primary antibodies (ZSGB-BIO, China). The blots were visualized with an enhanced chemiluminescence (ECL) detection system (Thermo Scientific, USA).

**Immunohistochemical staining**

Following deparaffinization and rehydration, tissue samples (5mm slices) were incubated in 0.3% H_2_O_2_ for 30 min at room temperature to block endogenous peroxidase. After PBS cleaning and antigen repair with 10 mmol/L citrate buffer (pH 6.0), tissue samples were incubated with anti-CPS1, MMP1 (Abcam, UK), CCL5, ALDH1A3 (Proteintech, China) overnight at 4 °C. CPS1 was detected using HRP-conjugated anti-rabbit secondary antibody (ZSGB-BIO, China) and visualized with DAB. The negative control was incubated only with the secondary antibody. The results were verified by two pathologists independently.

**Immunofluorescence assay**

Cells were fixed with 4% polyformaldehyde for 30 min, and blocked with goat serum for 1 h at room temperature. Cells were incubated with an anti-CPS1 or anti-EpCAM antibody for 1 h at room temperature. After three washes in PBS, cells were incubated with a Cy3-conjugated or FITC-conjugated secondary antibody (Beyotime, China) for 1 h. The nuclei were stained with DAPI for 5 min. Images were captured on a confocal microscope (Leica SP5, Germany).

**Supplementary Table 1** Sequences of all used oligos

| Primer sequence | | |
| --- | --- | --- |
| CPS1 | Forward | ACTGAAGGCAGCAGACACCATTG |
| CPS1 | Reverse | CCAACCTGTCACTGACTTCTCCAC |
| SLC1A3 | Forward | ATGAGGCTTTGGCTGCCATTTT |
| SLC1A3 | Reverse | AGGAATTCCAGCTGCCCCAATA |
| MMP1 | Forward | AGATTCTACATGCGCACAAATC |
| MMP1 | Reverse | CCTTTGAAAAACCGGACTTCAT |
| ALDH1A3 | Forward | CTTCAACTCGGGAGCAAATATG |
| ALDH1A3 | Reverse | TGAAGAAATGGCTTCCCTGTAT |
| CCL5 | Forward | ATTTGCCTGTTTCTGCTTGCTCTTG |
| CCL5 | Reverse | AACTGCTGCTGTGTGGTAGAATCTG |
| METTL-14 | Forward | GGCTATGACTCCTAATCACGCTTCC |
| METTL-14 | Reverse | ATCCAAGTTCAAGTCCACACCACAG |
| METTL3 | Forward | CTTCAGCAGTTCCTGAATTAGC |
| METTL3 | Reverse | ATGTTAAGGCCAGATCAGAGAG |
| WTAP | Forward | CTGACAAACGGACCAAGTAATG |
| WTAP | Reverse | AAAGTCATCTTCGGTTGTGTTG |
| BRM15B | Forward | ATCTTTCAGAGTACGCTCAGAC |
| BRM15B | Reverse | CTAGGATATGCATAGACGTGGG |
| VIRMA | Forward | GGAATGGACACGTTTATTCGAG |
| VIRMA | Reverse | GATAGAGCACAGGAGCATATGT |
| ZC3H13 | Forward | GATCAGTTAAAGCGTGGAGAAC |
| ZC3H13 | Reverse | CTCTCTGTCGTGTTCATATCGA |
| METTL16 | Forward | AGTACCATCACCACCAAGTAAG |
| METTL16 | Reverse | TTTCAATCCATGTCGTGACAAC |
| β-Actin | Forward | GGCATCCACGAAACTACCTT |
| β-Actin | Reverse | CGGACTCGTCATACTCCTGCT |
| Interference sequence | | |
| shCPS1-640 | GCTGGCTACCAAGAGTTTAGG  CGAACCTAAACTCTTGGTAGCCAGC | |
| shCPS1-641 | GCCCTTCATCCTACCTCAATGC  GAACATTGAGGTAGGATGAAGGGC | |
| shCPS1-642 | GCTGACTATGTTGCAATTAAG  CGAACTTAATTGCAACATAGTCAGC | |
| siMETTL-14-240 | Sense | GCCGUGUUAAAUAGCAAAGAUTT |
|  | Antisense | AUCUUUGCUAUUUAACACGGCTT |
| siMETTL-14-643 | Sense | CCAUGUACUUACAAGCCGAUATT |
|  | Antisense | UAUCGGCUUGUAAGUACAUGGTT |
| siMETTL-14-1414 | Sense | GCCGUGGACGAGAAAGAAAUATT |
|  | Antisense | UAUUUCUUUCUCGUCCACGGCTT |
| siMETTL3-880 | Sense | GCCAAGGAACAAUCCAUUGUUTT |
|  | Antisense | AACAAUGGAUUGUUCCUUGGCTT |
| siMETTL3-1094 | Sense | GCAAGUAUGUUCACUAUGAAATT |
|  | Antisense | UUUCAUAGUGAACAUACUUGCTT |
| siMETTL3-385 | Sense | GCCUUAACAUUGCCCACUGAUTT |
|  | Antisense | AUCAGUGGGCAAUGUUAAGGCTT |
| siWTAP-427 | Sense | GCAAGAGUGUACUACUCAAAUTT |
|  | Antisense | AUUUGAGUAGUACACUCUUGCTT |
| siWTAP-1176 | Sense | GGAGGUAGUGGUUACGUAAAUTT |
|  | Antisense | AUUUACGUAACCACUACCUCCTT |
| siWTAP-637 | Sense | CCUGGAAGUUUACGCCUGAUATT |
|  | Antisense | UAUCAGGCGUAAACUUCCAGGTT |
| siNC | Sense | UUCUCCGAACGUGUCACGUTT |
|  | Antisense | ACGUGACACGUUCGGAGAATT |

Primers or interference sequences in this study.

**Supplementary table 2.** The molecular docking results for the binding between amino acids and yeast PC-PLC

| Target | Compd | Docking Score (kcal/mol) | Combination Type |
| --- | --- | --- | --- |
| PC-PLC | Asp | -6.79 | Hydrogen bonds, Electrostatic interaction  Hydrophobic interactive |
|  | Arg | -6.56 | Hydrogen bonds, Electrostatic interaction  Hydrophobic interactive |
|  | Orn | -6.32 | Hydrogen bonds, Electrostatic interaction  Hydrophobic interactive |

Asp: Aspartate, Arg: Arginine, Orn: Ornithic acid.

**Supplementary Figures**


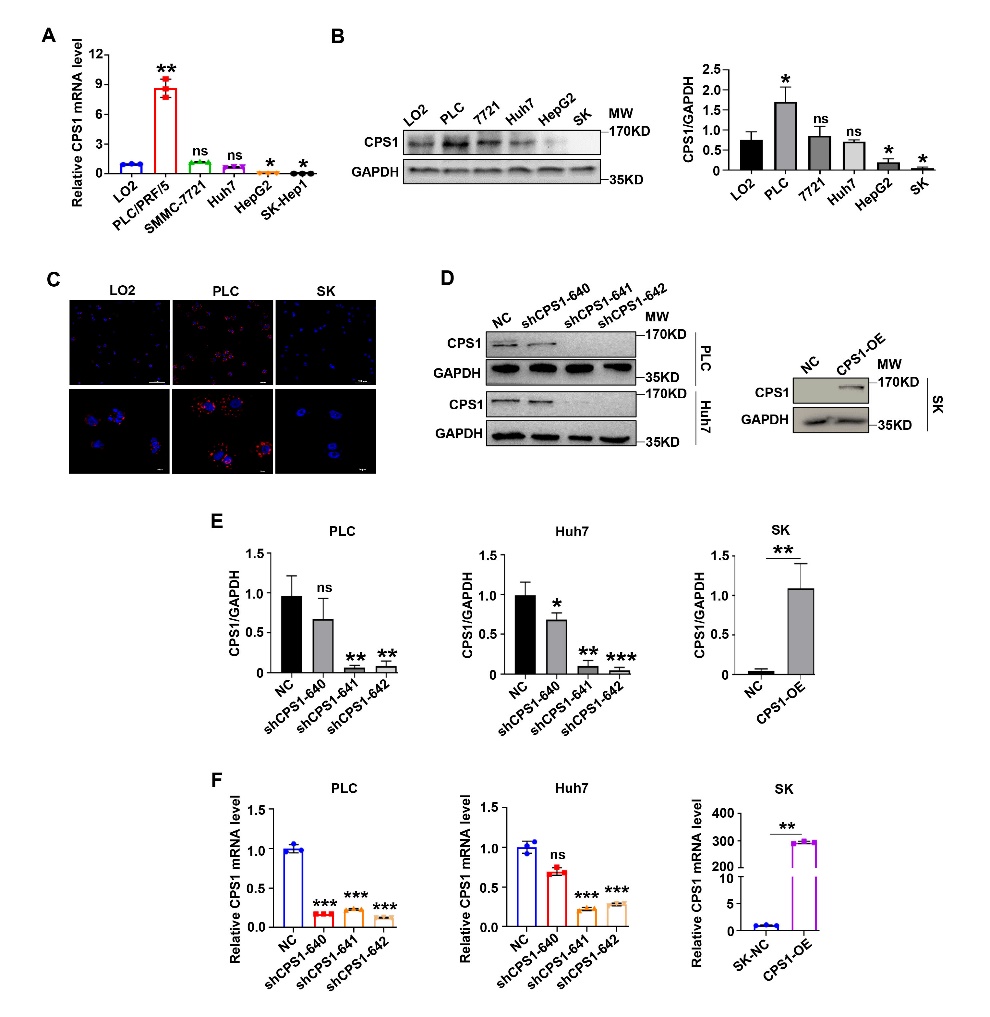


**Supplementary Figure 1. Expression and screening of CPS1 in different liver cancer cell lines.** A) qPCR detection of CPS1 mRNA levels in different liver cancer cell lines and normal liver cell line. B) WB detection of CPS1 protein expression levels in different liver cancer cells. PLC/PRF/5: PLC, SMMC-7721: 7721, SK-Hep1: SK. The left panel is the WB image, the right panel is grayscale statistics of protein expression values. C) Immunofluorescence detection of CPS1 expression and subcellular localization. The scale bars on the lower right are 100 µm (upper panel) and 20 µm (lower panel). D) WB was used to detect the knockdown efficiency of CPS1 interfering lentivirus in PLC and Huh7 cells, and the overexpression efficiency of CPS1 overexpressing adenovirus in SK cells. E) Grayscale statistics of CPS1 protein expression. F) qPCR was used to detect the knockdown efficiency of CPS1 interfering lentivirus in PLC and Huh7 cells, and the overexpression efficiency of CPS1 overexpressing adenovirus in SK cells. *: p<0.05, **: p<0.01, ***: p<0.001, ns: no significant difference. The above experiments have all been biologically replicated (n = 3).


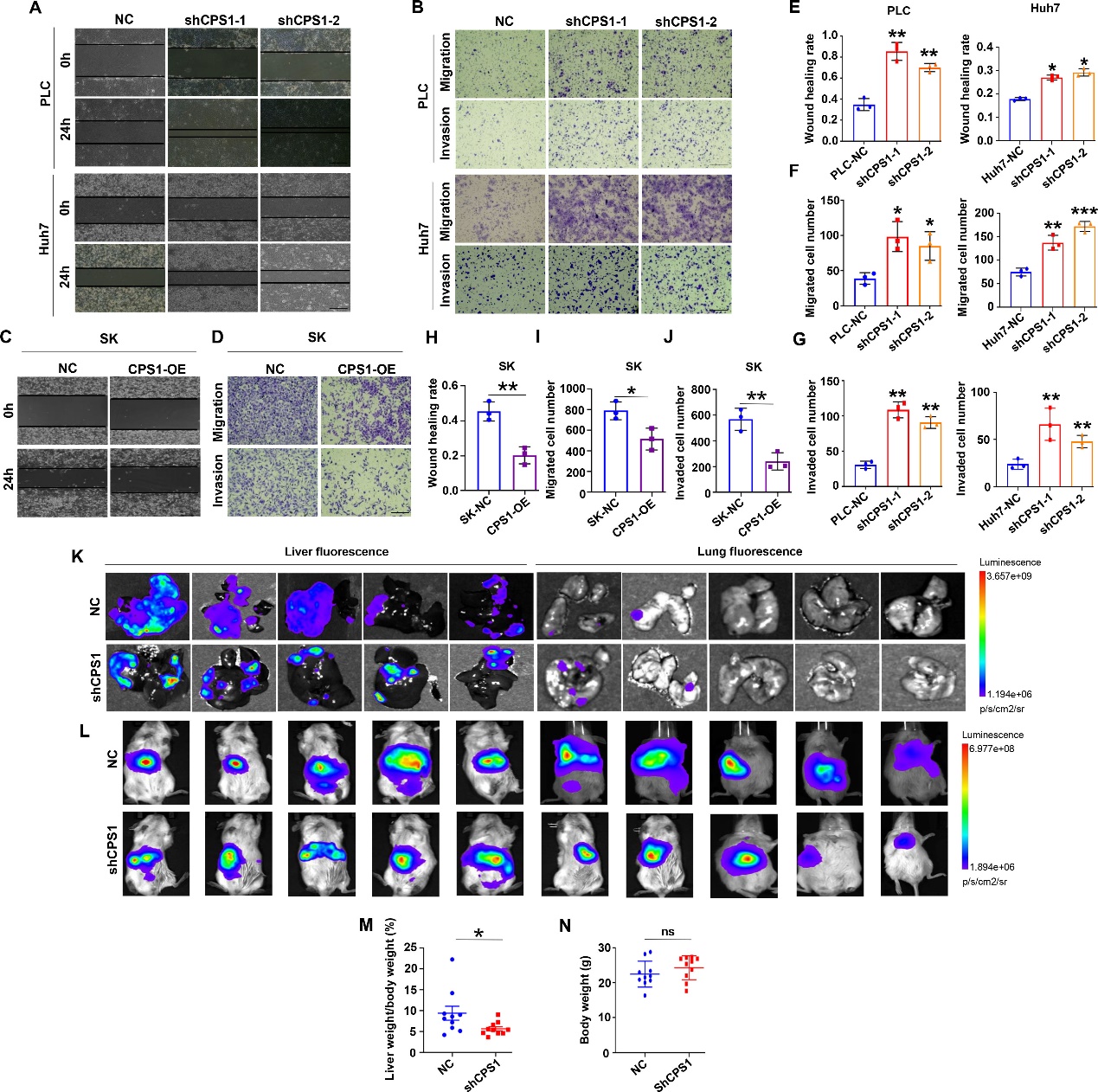


**Supplementary Figure 2.** **The regulatory effect of CPS1 on the invasion and migration of HCC cells.** A&E) Wound healing experiment detected migration of PLC-NC, PLC-shCPS1, Huh7-NC, Huh7-shCPS1. B&F&G) Transwell assay on the effect of CPS1 on migration and invasion in PLC, Huh7 cells. C&H) Wound healing experiment detected migration of SK-NC and SK-CPS1-OE cells. D&I&J) Transwell assay on the effect of CPS1 on migration and invasion in SK-NC and SK-CPS1-OE cells. K&L) Fluorescence imaging was used to detect in situ tumorigenic mice and their livers or lungs. M) Statistics of liver weight ratio in situ tumorigenic mice. N) Body weight statistics of in situ tumorigenic mice. Scale bars are 200 µm, *: p<0.05, **: p<0.01, ***: p<0.001. The above in vitro experiments have all been biologically replicated (n = 3).


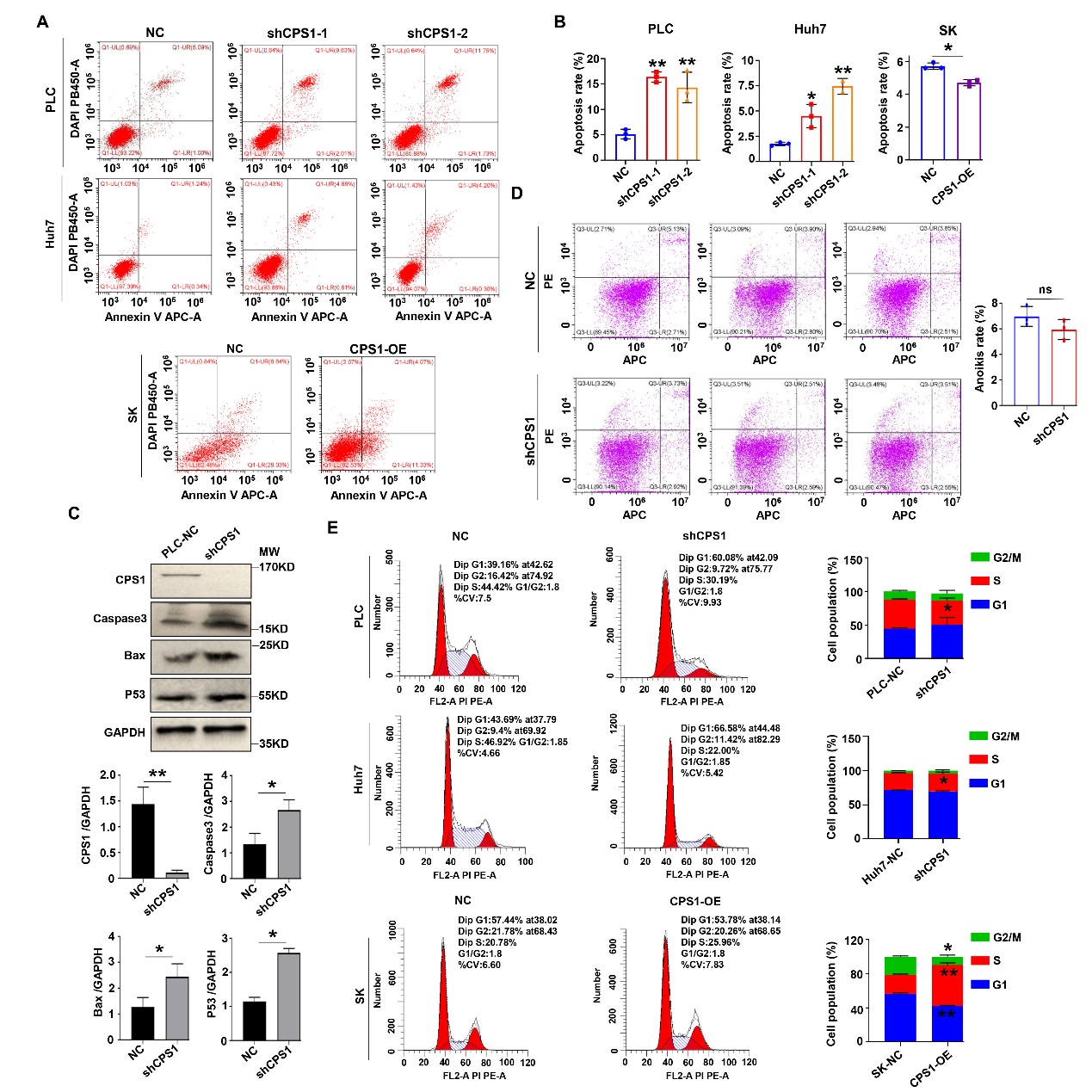


**Supplementary Figure 3.** **The effect of CPS1 on apoptosis/cell cycle of HCC cells.** A) Flow cytometry analysis of changes in cell apoptosis after knocking down or overexpressing CPS1. B) Statistical chart of cell apoptosis. C) WB detected the changes in protein levels of apoptosis related genes in PLC-NC and PLC-shCPS1 cells. The upper panel is the WB image, the lower panel the grayscale statistics of protein expression values. D) Flow cytometry detection of anoikis in PLC-NC and PLC-shCPS1 cells. The left panels are the anoikis images, the right panel is statistical chart of cell anoikis. E) Changes in the number of G1, S, G2/M phase cells after knocking down or overexpressing CPS1, detected by flow cytometry. The left panels are the cell cycle phase images, the right panel is statistical chart of all phases. *: p<0.05, **: p<0.01, ns: no significant difference. The above experiments have all been biologically replicated (n = 3).


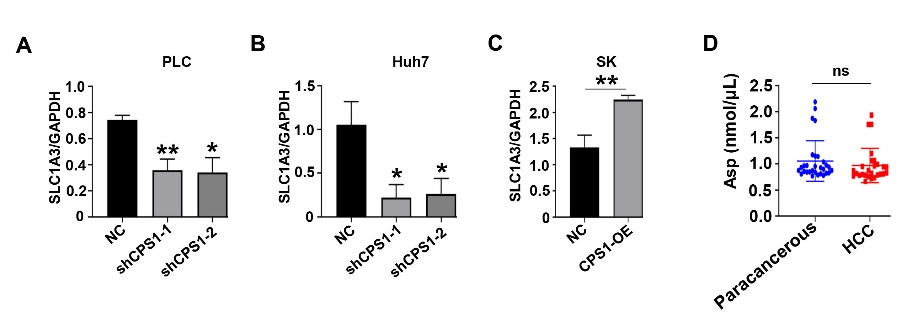


**Supplementary Figure 4.** **Regulation of SLC1A3/Asp by CPS1.** A-C) Grayscale statistical analysis of protein expression of SLC1A3 in PLC-NC, PLC-shCPS1, Huh7-NC, Huh7-shCPS1, SK-NC and SK-CPS1-OE cells. D) The content of Aspartate (Asp) in the serum of HCC patients and normal individuals, n=20. *: p<0.05, **: p<0.01, ns: no significant difference.


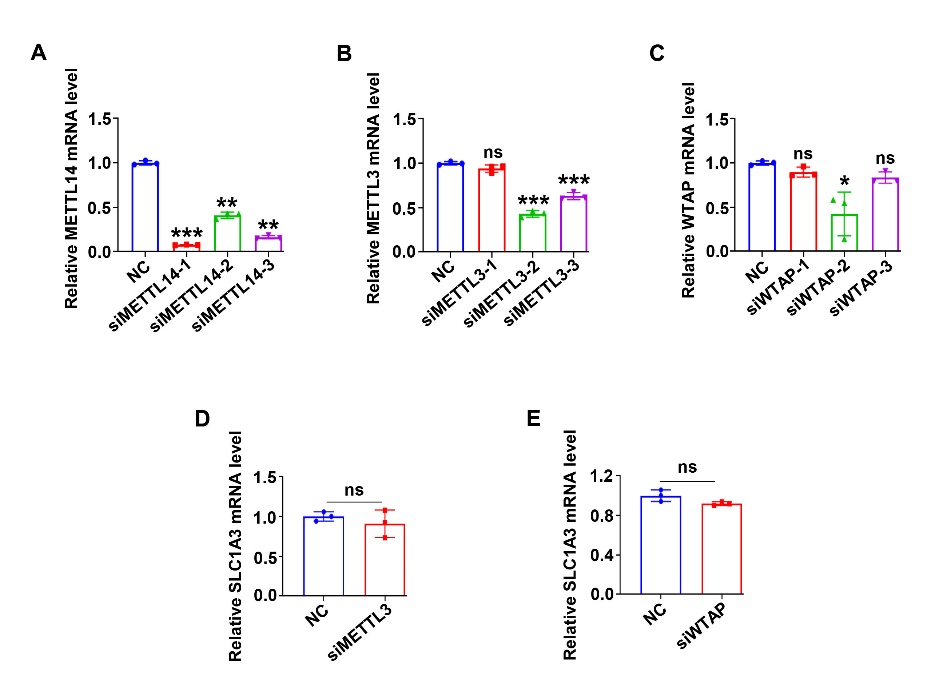


**Supplementary Figure 5.** **Screening m6A writers that regulate SLC1A3 expression.** A-C) qPCR detection of knockdown efficiency of siRNA on METTL14, METTL3, and WTAP. D-E) qPCR detected the effect of METTL3 and WTAP on SLC1A3 mRNA level. *: p<0.05, **: p<0.01, ***: p<0.001, ns: no significant difference. The above experiments have all been biologically replicated (n = 3).


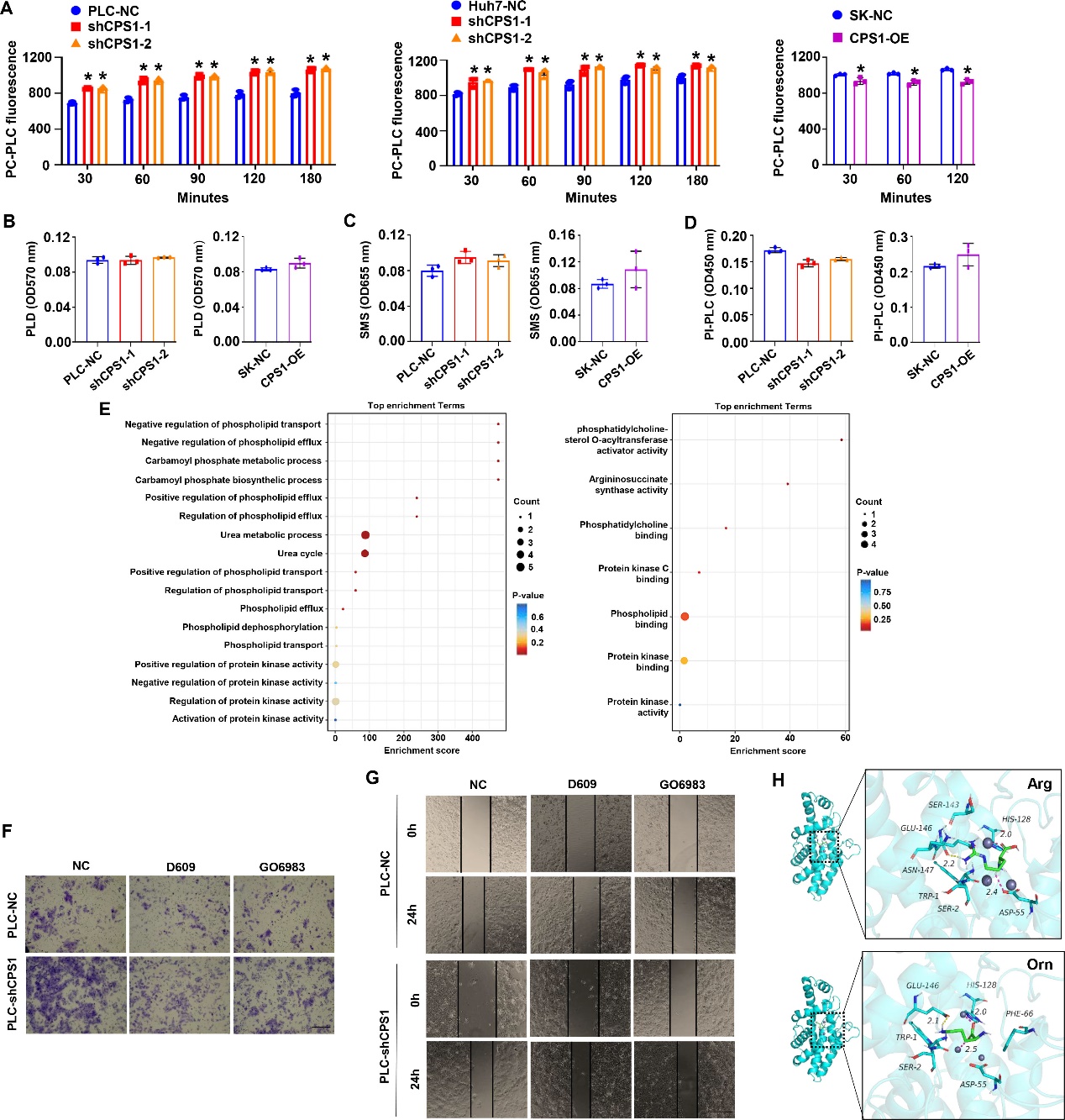


**Supplementary Figure 6.** **Changes of key enzymes involved in DAG production.** A) Detection of PC-PLC activity in PLC-shCPS1, Huh7-shCPS1, SK-CPS1-OE and corresponding NC cells. B) The effect of CPS1 on phospholipase D (PLD) activity. C) Regulation of Sphingomyelin synthase (SMS) activity by CPS1. D) Effect of CPS1 on phosphatidylinositol specific phospholipase C (PI-PLC) activity. E) GO analysis of changes in metabolic pathways. F&G) Images show the effect of PC-PLC inhibitor D609 and PKC inhibitor GO6983 on the invasion and migration of PLC-NC/shCPS1 cells detected by transwell and wound healing experiment. H) Molecular docking analysis of binding sites between PC-PLC and Arginine (Arg) and Ornithic acid (Orn). Scale bars are 200 µm, *: p<0.05.


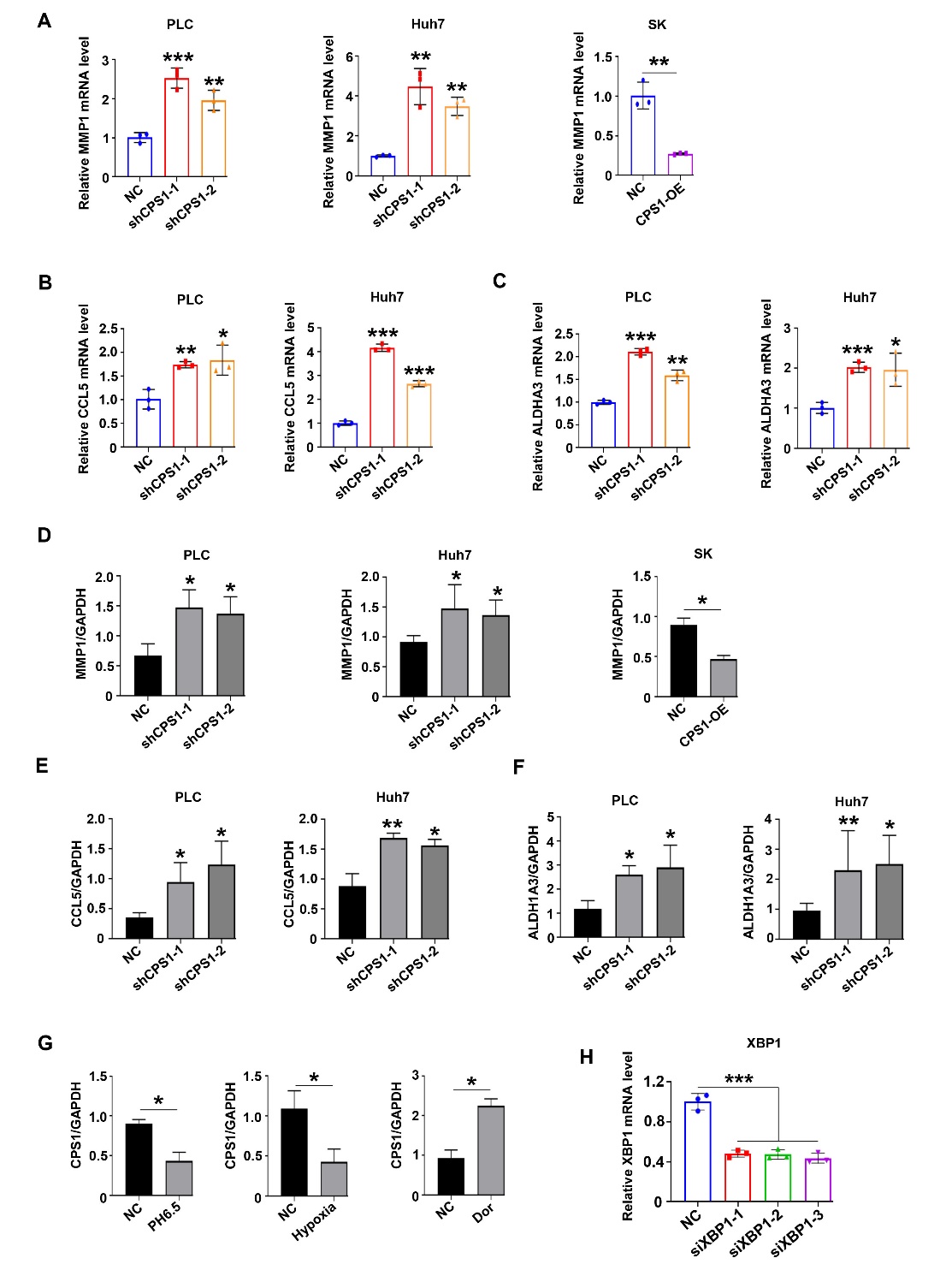


**Supplementary Figure 7.** **Upstream and downstream regulation of CPS1.** A) qPCR detection of changes in mRNA levels of MMP1 in PLC-NC/shCPS1, Huh7-NC/shCPS1, SK-NC/CPS1-OE cells. B) qPCR detection of the effect of CPS1 on CCL5 mRNA level. C) qPCR detection of the effect of CPS1 on ALDH1A3 mRNA level. D-F) Grayscale Statistics of the regulation of MMP1/CCL5/ALDH1A3 by CPS1. G) Grayscale statistical analysis of protein level changes in CPS1 after PH6.5, hypoxia, and AMPK inhibitor (Dorsomorphin dihydrochloride) treatment. H) qPCR detection of knockdown efficiency of XBP1 by siRNA. *: p<0.05, **: p<0.01, ***: p<0.001. The above experiments have all been biologically replicated (n = 3).
